# National trends and demographic disparities in mortality involving co-listed chronic obstructive pulmonary disease and substance use disorders among U.S. adults aged ≥65 years: A population-based serial cross-sectional CDC WONDER analysis, 1999 to 2020

**DOI:** 10.1097/MD.0000000000051448

**Published:** 2026-11-06

**Authors:** Mirza Ammar Arshad, Urwah Noor, Rida Fatima, Fatima Maqsood, Hassan Faraz Chaudhry, Muhammad Shumas, Amna Zafar, Menahil Fazal, Ravi Kumar Pandey, Rida Khan, Muhammad Hassan, Ashish Kumar, Abdullah Naeem, Usman Zia, Yahye Ahmed Nor

**Affiliations:** a Department of Medicine, Rahbar Medical & Dental College, Lahore, Pakistan; b Department of Medicine, FMH College of Medicine & Dentistry, Lahore, Pakistan; c Department of Medicine, Punjab Medical College, Faisalabad, Pakistan; d Department of Medicine, Islam Medical and Dental College, Sialkot, Pakistan; e Department of Medicine, Rashid Latif Medical College, Lahore, Pakistan; f Department of Medicine, Akhtar Saeed Medical & Dental College, Lahore, Pakistan; g Department of Medicine, Lahore Medical & Dental College, Lahore, Pakistan; h Department of Medicine, Allama Iqbal Medical College, Lahore, Pakistan; i Department of Internal Medicine, Nepalgunj Medical College, Nepalgunj, Nepal; j Department of Medicine, Ziauddin Medical College, Karachi, Pakistan; k Department of Medicine, Shalamar Medical and Dental College, Lahore, Pakistan; l Department of Medicine, Dow International Medical College, Karachi, Pakistan; m Department of Medicine, Shalamar Medical and Dental College, Lahore, Pakistan; n Department of Medicine, Akhtar Saeed Medical & Dental College, Lahore, Pakistan; o Faculty of Medicine and Surgery, Salaam University, Mogadishu, Somalia; p Faculty of Medicine and Health Science, Somali National University, Mogadishu, Somalia.

**Keywords:** CDC WONDER, chronic obstructive pulmonary disease, epidemiology, health disparities, mortality, substance use disorder

## Abstract

Chronic obstructive pulmonary disease (COPD) and substance use disorders (SUDs) frequently coexist, yet national mortality patterns involving both conditions among older adults remain incompletely characterized. We evaluated temporal, demographic, geographic, and substance-specific patterns in U.S. death records co-listing COPD and SUDs among adults aged ≥65 years. We conducted a descriptive serial cross-sectional analysis of Centers for Disease Control and Prevention Wide-ranging Online Data for Epidemiologic Research multiple cause of death data from 1999 to 2020. Eligible records included deaths among adults aged ≥65 years in which COPD (International Classification of Diseases, 10th revision J41–J44) and SUDs (F10–F19) were both recorded in the Multiple Cause of Death fields. Age-adjusted mortality rates (AAMRs) were calculated overall and by sex, race/ethnicity, census region, urbanization, and state; age-group analyses used age-specific crude mortality rates. Joinpoint regression estimated annual percent changes and average annual percent changes. Secondary analyses examined individual F10 to F19 categories, with a sensitivity analysis excluding tobacco/nicotine-related disorders (F17). Among 1,524,216 qualifying deaths, the overall AAMR increased from 25.5/100,000 in 1999 to 213.2 in 2020, peaking at 219.0 in 2017. Males had higher pooled AAMRs than females (216.98 vs 132.54). The highest pooled racial/ethnic AAMR occurred among non-Hispanic American Indian or Alaska Native adults (191.99), followed closely by non-Hispanic White adults (188.68). The Midwest had the highest regional AAMR, while nonmetropolitan counties had higher AAMRs than metropolitan counties (232.82 vs 152.03). Tobacco/nicotine-related disorders accounted for the predominant substance-specific burden. Excluding F17 yielded an average annual percent change of 2.19% (95% confidence interval, 1.37–3.02). In the overall F10 to F19 analysis, mortality involving co-listed COPD and SUDs increased markedly during the earlier study period before stabilizing, whereas temporal trajectories differed across individual substance categories. Substantial demographic, geographic, and substance-specific heterogeneity was observed. Findings are descriptive and should not be interpreted causally.

## Graphical abstract

**Figure:**
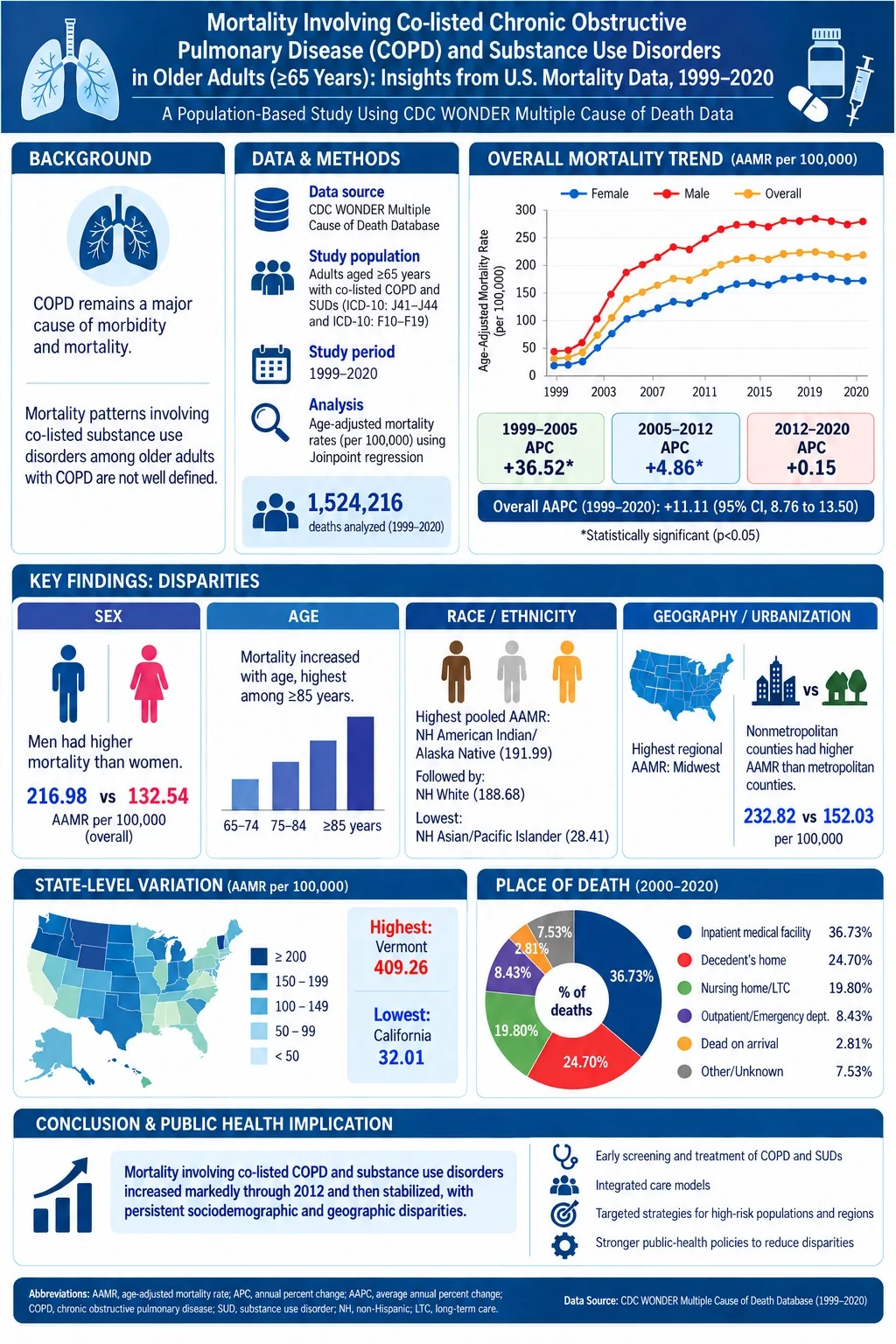

## 1. Introduction

Chronic obstructive pulmonary disease (COPD) remains a major global health burden and a leading cause of morbidity and mortality. In 2019, it was the 3rd leading cause of death worldwide, accounting for approximately 3.23 million deaths.^[1]^ In the United States, chronic lower respiratory diseases, predominantly COPD, remain a major cause of death, and >14 million Americans are estimated to live with COPD.^[2,3]^ Substance use disorders (SUDs) also represent an important public health concern, particularly because substance exposure and chronic respiratory disease frequently coexist in populations with substantial comorbidity.^[4,5]^

Several exposures encompassed by SUD diagnoses may be clinically relevant to COPD. Tobacco smoking is the principal established risk factor for COPD and remains a major preventable cause of disease and death in the United States.^[6]^ Alcohol, cannabis, opioids, and other substances may also coexist with respiratory disease and can be associated with medication nonadherence, respiratory depression, recurrent health-care utilization, or other adverse outcomes.^[5]^ However, these relationships are heterogeneous across substances, and death-certificate data cannot determine whether an SUD caused, worsened, or merely co-occurred with COPD in an individual decedent.

Prior epidemiologic studies have described long-term COPD mortality trends in the United States, including differences by sex, race, geography, and urbanization.^[7–9]^ Other studies have examined substance use in COPD populations or substance-use-related mortality in different disease settings.^[4,10–12]^ Nevertheless, national mortality patterns specifically among older adults whose death certificates co-list both COPD and SUDs have not been well characterized. This distinction is relevant because adults aged ≥65 years have a high COPD burden, substantial multimorbidity, and unique vulnerabilities related to polypharmacy, frailty, and access to pulmonary and addiction care.^[3,13,14]^

Accordingly, we used the Centers for Disease Control and Prevention Wide-ranging Online Data for Epidemiologic Research (CDC WONDER) Multiple Cause of Death database to describe national temporal trends and demographic and geographic disparities in death records co-listing COPD and SUDs among U.S. adults aged ≥65 years from 1999 through 2020. We examined patterns by sex, race/ethnicity, age group, census region, urbanization, and state and used Joinpoint regression to characterize changes in mortality rates over time. The analysis was designed to identify population groups and geographic settings with comparatively high mortality burden while avoiding causal interpretation of the co-listed diagnoses.

## 2. Methods

### 2.1. Study setting and population

We conducted a descriptive, population-based serial cross-sectional study using death-certificate data from the CDC WONDER Multiple Cause of Death database for 1999 to 2020. Eligible records were deaths among adults aged ≥65 years in which both COPD (International Classification of Diseases, 10th revision codes J41–J44) and SUDs (International Classification of Diseases, 10th revision codes F10–F19) were recorded in the Multiple Cause of Death fields. Neither condition was required to be the underlying cause of death. Population denominators were derived from the corresponding U.S. resident population aged ≥65 years for each calendar year and subgroup, as provided by CDC WONDER, rather than from a population of individuals known to have COPD or SUDs.

Accordingly, the analytic population comprised decedents whose death certificates co-listed COPD and SUDs rather than a clinical cohort of patients with confirmed COPD and SUD diagnoses. Thus, the analysis characterizes mortality involving co-listed COPD and SUDs but cannot determine which condition, if either, was the underlying cause of death or establish the temporal or causal relationship between them.

To address heterogeneity within the F10 to F19 category, we performed a secondary substance-specific analysis examining alcohol-related disorders (F10), opioid-related disorders (F11), cannabinoid-related disorders (F12), sedative/hypnotic-related disorders (F13), cocaine-related disorders (F14), other stimulant-related disorders (F15), hallucinogen-related disorders (F16), tobacco/nicotine-related disorders (F17), volatile solvent-related disorders (F18), and multiple/other psychoactive substance-related disorders (F19). Substance-specific categories were not considered mutually exclusive because >1 substance-related diagnosis could be recorded on the same death certificate. Estimates designated as unreliable by CDC WONDER were retained for descriptive reporting but excluded from trend modeling, whereas suppressed estimates were not reconstructed. Joinpoint regression was performed only for substance categories with complete and reliable annual age-adjusted mortality rate (AAMR) series across 1999 to 2020.

Because tobacco/nicotine-related disorders (F17) may share a particularly strong epidemiologic relationship with COPD through smoking exposure, we additionally performed a sensitivity analysis, repeating the overall mortality analysis after excluding F17 from the F10 to F19 definition. All other eligibility criteria, mortality-rate calculations, and Joinpoint procedures were unchanged.

The dataset includes mortality records from all 50 states and the District of Columbia. Adults aged ≥65 years were selected a priori to represent the older-adult population, consistent with commonly used U.S. epidemiologic definitions and prior studies focused on older adults with COPD.^[3,15,16]^ Because CDC WONDER provides publicly available, de-identified aggregate data, institutional review board approval and informed consent were not required. The study was reported in accordance with the Strengthening the Reporting of Observational Studies in Epidemiology guidelines.^[17]^

### 2.2. Data abstractions

We abstracted calendar year, sex, 10-year age group, race/ethnicity, U.S. census region, state, and urbanization. Race/ethnicity was categorized into mutually exclusive groups as non-Hispanic (NH) White, NH Black or African American, NH American Indian or Alaska Native, NH Asian or Pacific Islander, and Hispanic or Latino, consistent with CDC WONDER classifications.^[18]^ Non-Hispanic racial groups were restricted to records classified as not Hispanic or Latino, whereas Hispanic or Latino mortality was extracted separately.

Urbanization was defined using the 2013 National Center for Health Statistics Urban–Rural Classification Scheme. Metropolitan counties were those within a Metropolitan Statistical Area, whereas nonmetropolitan counties included micropolitan and noncore counties.^[7,19,20]^ Census regions were classified as Northeast, Midwest, South, and West, according to U.S. Census Bureau definitions. All subgroup variables were analyzed as recorded in the aggregate database; no individual-level clinical or socioeconomic covariates were available.

### 2.3. Statistical analysis

Crude mortality rates were calculated by dividing the number of qualifying deaths by the corresponding U.S. resident population within the same calendar year and demographic or geographic stratum and multiplying by 100,000. Thus, the denominator represented the general U.S. population aged ≥65 years within the relevant stratum rather than the number of individuals diagnosed with COPD, SUDs, or both conditions. Age-group analyses were reported as age-specific crude mortality rates. For the overall population and analyses stratified by sex, race/ethnicity, census region, urbanization, and state, AAMRs were standardized to the 2000 U.S. standard population and reported per 100,000 population with 95% confidence intervals (CIs).

Temporal trends were evaluated using the Joinpoint Regression Program version 6.1.0 (National Cancer Institute [NCI]).^[21]^ Log-linear Joinpoint regression models were fitted to annual mortality rates using the Grid Search method. The number of joinpoints was allowed to range from 0 to 4. A minimum of 2 observations was required before the 1st joinpoint and after the last joinpoint, and at least 2 observations were required between adjacent joinpoints. No additional grid points were inserted between observed annual calendar-year values. The optimal number and location of joinpoints were selected using the permutation-test method with an overall significance level of 0.05 and 4499 permutations. Annual percent change (APC) was calculated for each fitted temporal segment, while average annual percent change (AAPC) summarized the average change across the entire 1999 to 2020 study period. Parametric 95% CIs were used for APC and AAPC estimates. Temporal trends were considered statistically significant when the corresponding 95% CI excluded zero.^[21]^

For the secondary substance-specific analysis, Joinpoint regression was performed only for alcohol-related disorders (F10), tobacco/nicotine-related disorders (F17), and multiple/other psychoactive substance-related disorders (F19), because these categories had complete and reliable annual AAMR series throughout 1999 to 2020. Categories with sparse, suppressed, unreliable, or otherwise incomplete annual estimates were not subjected to Joinpoint modeling because such series were considered unsuitable for stable longitudinal trend estimation. Suppressed values were not reconstructed or imputed. Accordingly, the remaining substance-specific categories were summarized descriptively. The sensitivity analysis excluding tobacco/nicotine-related disorders (F17) was evaluated using the same Joinpoint procedures as the primary overall analysis.^[21]^

Because CDC WONDER provides aggregate rather than individual-level data, multivariable adjustment for smoking exposure, COPD severity, socioeconomic conditions, comorbidities, health-care access, treatment, and other potential confounders was not possible. Accordingly, subgroup comparisons and temporal patterns were interpreted descriptively, and no causal inferences were made.

## 3. Results

### 3.1. Overall stratification

From 1999 to 2020, 1,524,216 death records co-listed COPD and SUDs (Table S1, Supplemental Digital Content 1). The overall AAMR increased from 25.5/100,000 (95% CI, 25.0–26.1) in 1999 to a peak of 219.0 (95% CI, 217.7–220.3) in 2017 and remained elevated at 213.2 (95% CI, 211.9–214.4) in 2020 (Table S2, Supplemental Digital Content 2). Mortality increased rapidly from 1999 to 2005 (APC, 36.52%; 95% CI, 27.74–45.90), followed by a slower increase from 2005 to 2012 (APC, 4.86%; 95% CI, 1.30–8.56). From 2012 to 2020, the trend was statistically stable (APC, 0.15%; 95% CI, −1.70 to 2.03). The overall AAPC was 11.11% (95% CI, 8.76–13.50) (Fig. 1; Table S2, Supplemental Digital Content 2).

**Figure 1.**
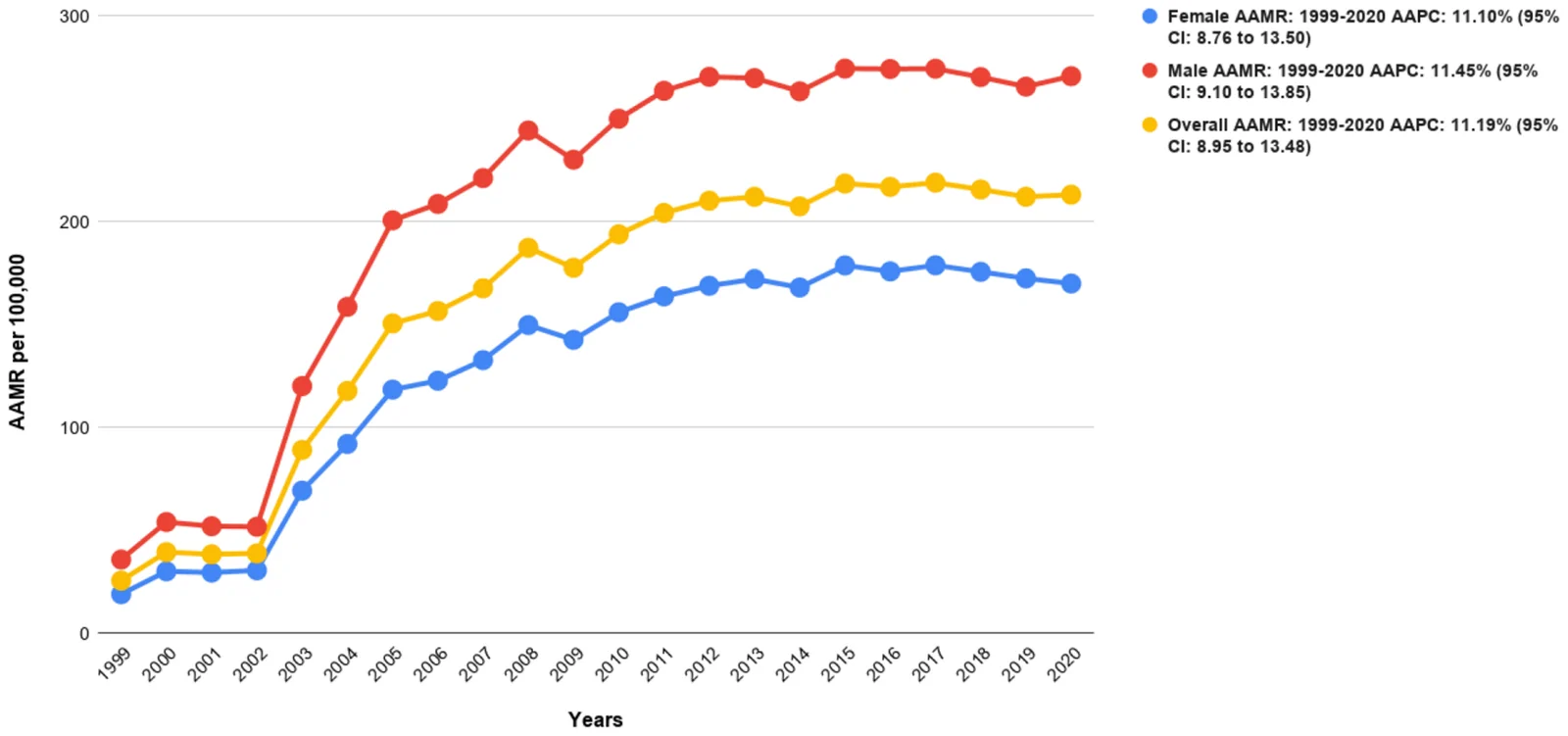
Chronic obstructive pulmonary disease and substance use disorder related AAMR per 100,000 by sex and overall, United States, 1999 to 2020. AAMR = age-adjusted mortality rate, AAPC = average annual percent change, CI = confidence interval.

### 3.2. Sex stratification

From 1999 to 2020, a total of 706,845 deaths among females and 817,371 deaths among males were identified among individuals with COPD and coexisting SUDs (Table S1, Supplemental Digital Content 1). Mortality was consistently higher in males than in females, both in absolute numbers and population-adjusted rates. Overall AAMRs were markedly greater in males (216.98; 95% CI, 216.51–217.46) than in females (132.54; 95% CI, 132.23–132.85) (Table S2, Supplemental Digital Content 2). The disparity was most evident in the mid-2010s, when the male AAMR peaked at 274.5/100,000 in 2015 compared with 178.8/100,000 among females. By 2020, mortality rates declined slightly for both sexes but remained substantially higher in males (270.76 vs 169.98/100,000) (Fig. 1).

Among females, mortality increased sharply between 1999 and 2005 (APC: 37.3%, *P* < .05), followed by a slower rise from 2005 to 2012 (APC: 5.3%, *P* < .05), and then stabilized thereafter (APC: 0.1%, *P* > .05), with an overall AAPC of 11.5% (95% CI, 9.1–13.9). In males, mortality rose rapidly from 1999 to 2005 (APC: 38.9%, *P* < .05) and continued to increase modestly through 2020 (APC: 1.7%, *P* < .05), yielding an overall AAPC of 11.2% (95% CI, 9.0–13.5) (Table 1). Although both sexes experienced steep early increases, female rates stabilized after 2012, whereas male rates continued to rise, maintaining a nearly 100-point higher AAMR by 2020 and underscoring a persistent sex-based disparity (Table S2, Supplemental Digital Content 2).

**Table 1:** Annual percent change (APC) in age-adjusted mortality rates (1999–2020) by different variables, highlighting temporal trends across key segments.

Cohort	Segment	Lower endpoint	Upper endpoint	APC	Lower CI	Upper CI	Overall AAPC
Overall	1	1999	2005	36.5159	27.7365	45.8987	11.1068 (8.7629–13.5002)
	2	2005	2012	4.8634	1.296	8.5565	
	3	2012	2020	0.1466	−1.6977	2.0254	
Female	1	1999	2005	37.3291	28.4751	46.7932	11.4556 (9.1032–13.8589)
	2	2005	2012	5.3367	1.7635	9.0354	
	3	2012	2020	0.1299	−1.7126	2.007	
Male	1	1999	2005	38.9221	29.0531	49.5459	11.1965 (8.9507–13.4887)
	2	2005	2020	1.723	0.8359	2.6179	
Northeast	1	1999	2006	43.5087	34.1731	53.4939	12.3334 (9.9116–14.8085)
	2	2006	2020	−0.6143	−1.6383	0.4205	
Midwest	1	1999	2008	27.821	22.8079	33.0387	11.8302 (9.0754–14.6544)
	2	2008	2012	5.5574	−4.8543	17.1085	
	3	2012	2020	−0.9635	−2.9513	1.0651	
South	1	1999	2005	35.5751	25.4383	46.531	11.1353 (8.7719–13.55)
	2	2005	2020	2.641	1.7095	3.581	
West	1	1999	2005	23.117	16.2667	30.3709	6.7806 (4.7387–8.8623)
	2	2005	2015	2.0046	0.0387	4.0092	
	3	2015	2020	−1.3614	−5.2765	2.7154	
American Indian or Alaska Native	1	1999	2005	34.9214	22.273	48.8783	9.8046 (6.8521–12.8386)
	2	2005	2020	1.1198	−0.0564	2.3098	
Asian or Pacific Islander	1	1999	2007	25.631	20.3926	31.0973	8.7979 (7.0803–10.5431)
	2	2007	2020	−0.4196	−1.2992	0.4679	
Black or African American	1	1999	2006	28.7407	23.0066	34.7421	11.1327 (9.4914–12.7985)
	2	2006	2020	3.2535	2.469	4.044	
White	1	1999	2005	37.0594	28.1806	46.5532	11.4862 (9.1183–13.9054)
	2	2005	2012	5.2787	1.6776	9.0074	
	3	2012	2020	0.3981	−1.469	2.3005	
Hispanic or Latino	1	1999	2005	32.3713	20.8034	45.047	8.7228 (6.0167–11.4981)
	2	2005	2020	0.492	−0.5321	1.5266	
Metropolitan	1	1999	2005	37.2983	28.48	46.7218	10.9742 (8.6312–13.3678)
	2	2005	2012	4.3021	0.7454	7.9842	
	3	2012	2020	−0.1259	−1.9959	1.7799	
Nonmetropolitan	1	1999	2004	41.5951	28.9916	55.43	12.2691 (9.6732–14.9263)
	2	2004	2012	7.9244	4.9289	11.0054	
	3	2012	2020	1.0202	−0.7937	2.8673	
65–74 yr	1	1999	2005	32.8868	24.666	41.6497	9.8857 (7.9497–11.8564)
	2	2005	2020	1.8415	1.0231	2.6667	
75–84 yr	1	1999	2005	38.2115	28.8488	48.2546	11.3783 (8.8852–13.9284)
	2	2005	2012	4.9601	1.175	8.8869	
	3	2012	2020	−0.2193	−2.2624	1.8665	
85+ yr	1	1999	2005	48.4169	36.608	61.2466	14.3190 (11.545–17.1619)
	2	2005	2013	5.2034	2.3929	8.0911	
	3	2013	2020	0.506	−1.7061	2.768	

AAPC = average annual percent change, APC = average percent change, CI = confidence interval.

### 3.3. Race/ethnicity stratification

From 1999 to 2020, AAMRs for death records co-listing COPD and SUDs varied substantially across racial and ethnic groups. The highest pooled AAMR was observed among NH American Indian or Alaska Native adults (191.99/100,000), followed closely by NH White adults (188.68), NH Black or African American adults (97.04), Hispanic or Latino adults (55.35), and NH Asian or Pacific Islander adults (28.41). In 2020, however, the highest AAMR was observed among NH White adults (248.24/100,000), followed by NH American Indian or Alaska Native adults (215.10), NH Black or African American adults (143.35), Hispanic or Latino adults (66.92), and NH Asian or Pacific Islander adults (33.85) (Fig. 2; Table S3, Supplemental Digital Content 3).

**Figure 2.**
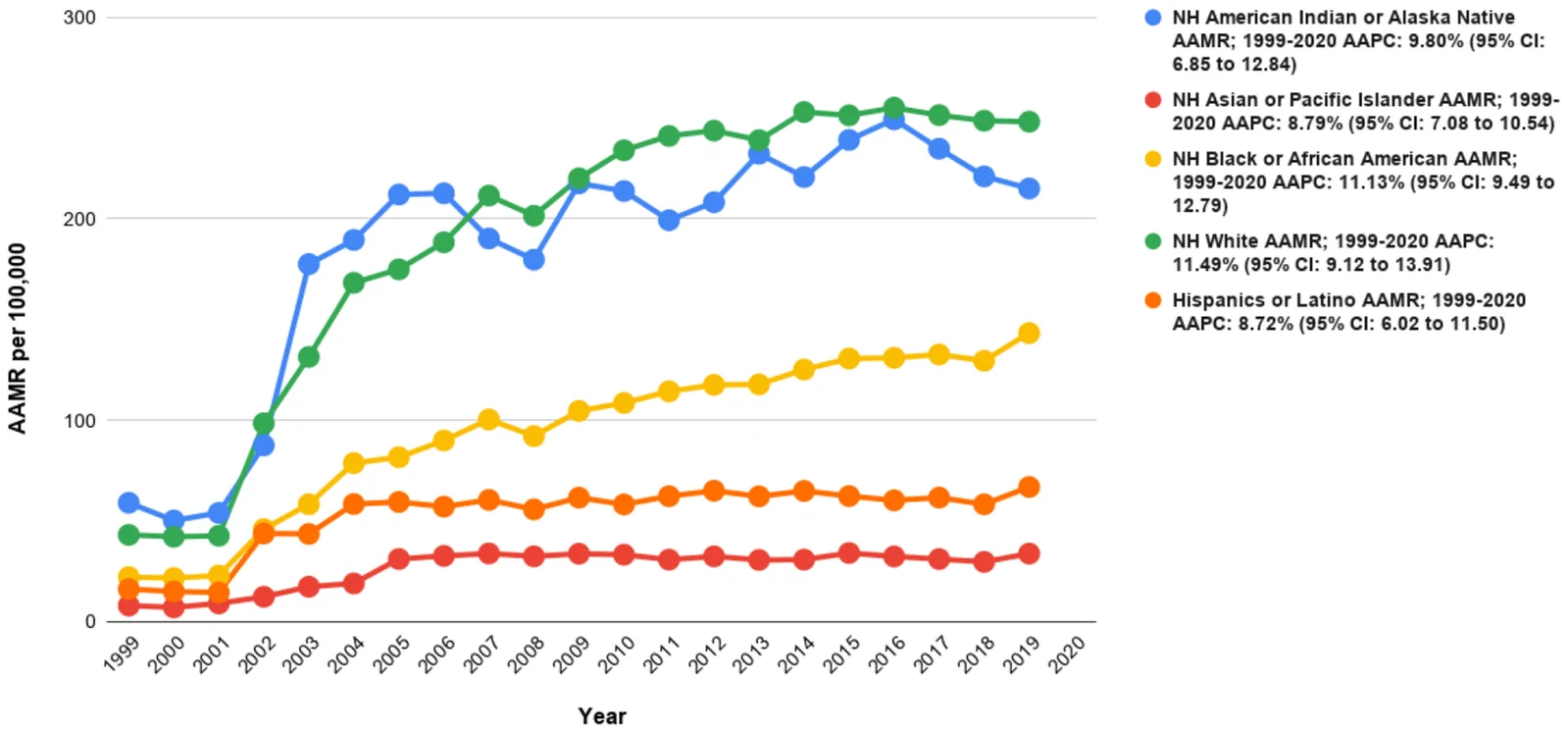
Chronic obstructive pulmonary disease and substance use disorder related AAMRs per 100,000 by race and ethnicity, United States, 1999 to 2020. AAMR = age-adjusted mortality rate, AAPC = average annual percent change, CI = confidence interval, NH = non-Hispanic.

Among NH American Indian or Alaska Native adults, mortality increased markedly from 1999 to 2005 (APC, 34.92%; 95% CI, 22.27–48.88) and was subsequently stable from 2005 to 2020 (APC, 1.12%; 95% CI, −0.06 to 2.31), yielding an overall AAPC of 9.80% (95% CI, 6.85–12.84). Among NH Asian or Pacific Islander adults, mortality increased from 1999 to 2007 (APC, 25.63%; 95% CI, 20.39–31.10) and remained stable thereafter (APC, −0.42%; 95% CI, −1.30 to 0.47). Among NH Black or African American adults, mortality increased from 1999 to 2006 (APC, 28.74%; 95% CI, 23.01–34.74) and continued to increase more gradually through 2020 (APC, 3.25%; 95% CI, 2.47–4.04). Among NH White adults, mortality increased sharply from 1999 to 2005 (APC, 37.06%; 95% CI, 28.18–46.55), increased more gradually from 2005 to 2012 (APC, 5.28%; 95% CI, 1.68–9.01), and was stable from 2012 to 2020 (APC, 0.40%; 95% CI, −1.47 to 2.30), yielding an overall AAPC of 11.49% (95% CI, 9.12–13.91). Hispanic or Latino adults showed an early increase from 1999 to 2005 (APC, 32.37%; 95% CI, 20.80–45.05), followed by a stable trend through 2020 (APC, 0.49%; 95% CI, −0.53 to 1.53) (Table 1).

### 3.4. Census region stratification

From 1999 to 2020, mortality among older adults with COPD and coexisting SUDs varied across U.S. census regions, with 539,930 deaths occurring in the South, 448,713 in the Midwest, 271,445 in the Northeast, and 264,128 in the West (Table S1, Supplemental Digital Content 1). Although the South accounted for the largest number of deaths, the highest pooled AAMR was observed in the Midwest (217.71/100,000), followed by the South (161.35), Northeast (150.11), and West (136.36) (Fig. 3; Table S5, Supplemental Digital Content 4).

**Figure 3.**
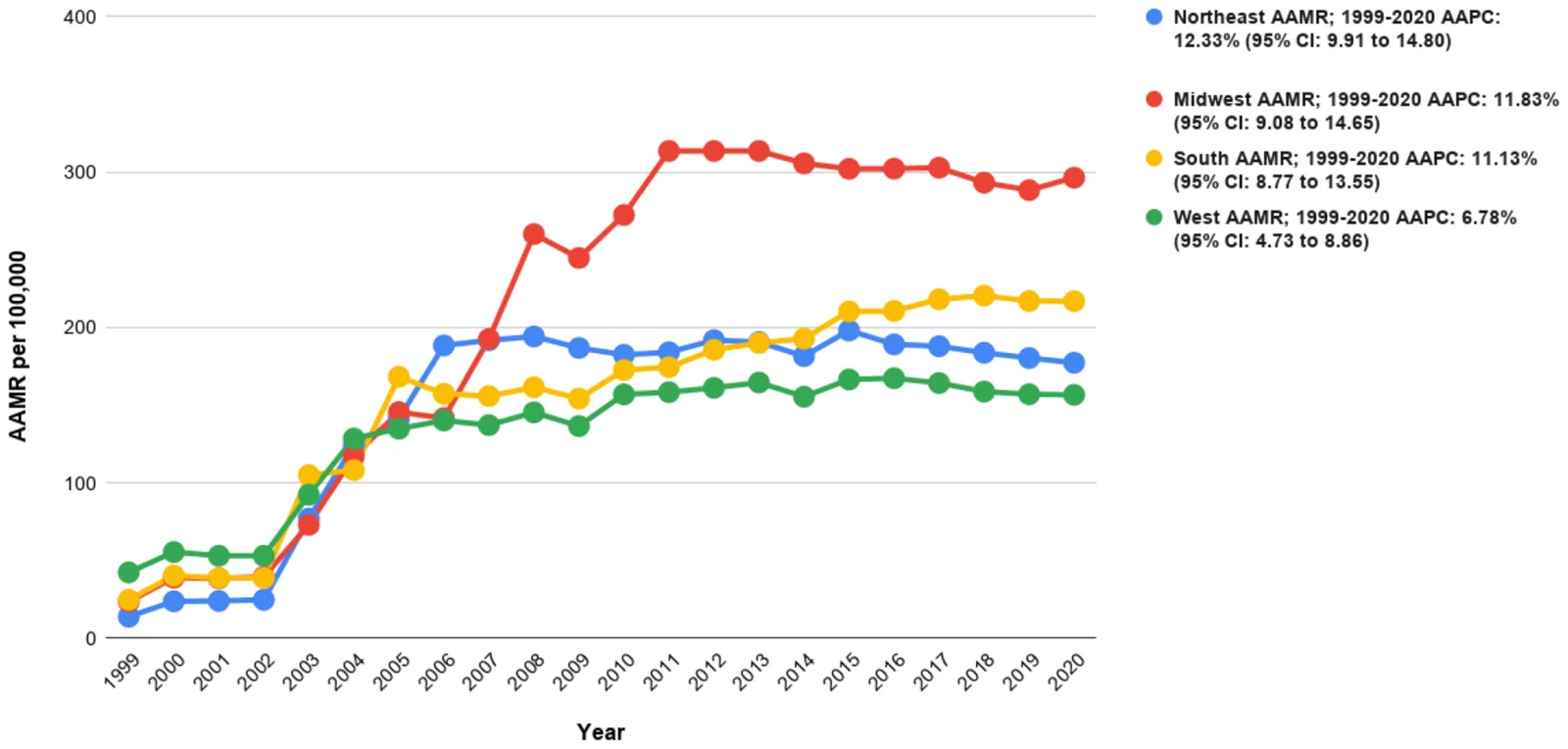
Chronic obstructive pulmonary disease and substance use disorder related AAMRs per 100,000 by census region, United States, 1999 to 2020. AAMR = age-adjusted mortality rate, AAPC = average annual percent change, CI = confidence interval.

In the Midwest, mortality increased from 1999 to 2008 (APC, 27.82%; 95% CI, 22.81–33.04), showed no statistically significant change from 2008 to 2012 (APC, 5.56%; 95% CI, −4.85 to 17.11), and remained stable from 2012 to 2020 (APC, −0.96%; 95% CI, −2.95 to 1.07), with an overall AAPC of 11.83% (95% CI, 9.08–14.65). In the South, mortality increased from 1999 to 2005 (APC, 35.58%; 95% CI, 25.44–46.53) and continued to increase from 2005 to 2020 (APC, 2.64%; 95% CI, 1.71–3.58), with an overall AAPC of 11.14% (95% CI, 8.77–13.55). In the Northeast, mortality increased from 1999 to 2006 (APC, 43.51%; 95% CI, 34.17–53.49) and was stable thereafter (APC, −0.61%; 95% CI, −1.64 to 0.42), yielding an overall AAPC of 12.33% (95% CI, 9.91–14.81). In the West, mortality increased from 1999 to 2005 (APC, 23.12%; 95% CI, 16.27–30.37), increased more modestly from 2005 to 2015 (APC, 2.00%; 95% CI, 0.04–4.01), and was stable from 2015 to 2020 (APC, −1.36%; 95% CI, −5.28 to 2.72), with an overall AAPC of 6.78% (95% CI, 4.74–8.86) (Table 1).

### 3.5. Age group stratification

From 1999 to 2020, age-specific mortality rates increased with advancing age. Among adults aged 65 to 74 years, mortality increased sharply from 1999 to 2005 (APC, 32.89%; 95% CI, 24.67–41.65) and continued to increase more gradually from 2005 to 2020 (APC, 1.84%; 95% CI, 1.02–2.67), yielding an overall AAPC of 9.89% (95% CI, 7.95–11.86). Among adults aged 75 to 84 years, mortality increased from 1999 to 2005 (APC, 38.21%; 95% CI, 28.85–48.25), increased more slowly from 2005 to 2012 (APC, 4.96%; 95% CI, 1.18–8.89), and was stable from 2012 to 2020 (APC, −0.22%; 95% CI, −2.26 to 1.87), with an overall AAPC of 11.38% (95% CI, 8.89–13.93). Among adults aged ≥85 years, mortality increased from 1999 to 2005 (APC, 48.42%; 95% CI, 36.61–61.25), increased more gradually from 2005 to 2013 (APC, 5.20%; 95% CI, 2.39–8.09), and remained stable from 2013 to 2020 (APC, 0.51%; 95% CI, −1.71 to 2.77), yielding an overall AAPC of 14.32% (95% CI, 11.55–17.16) (Fig. 4, Table 1; Table S8, Supplemental Digital Content 5).

**Figure 4.**
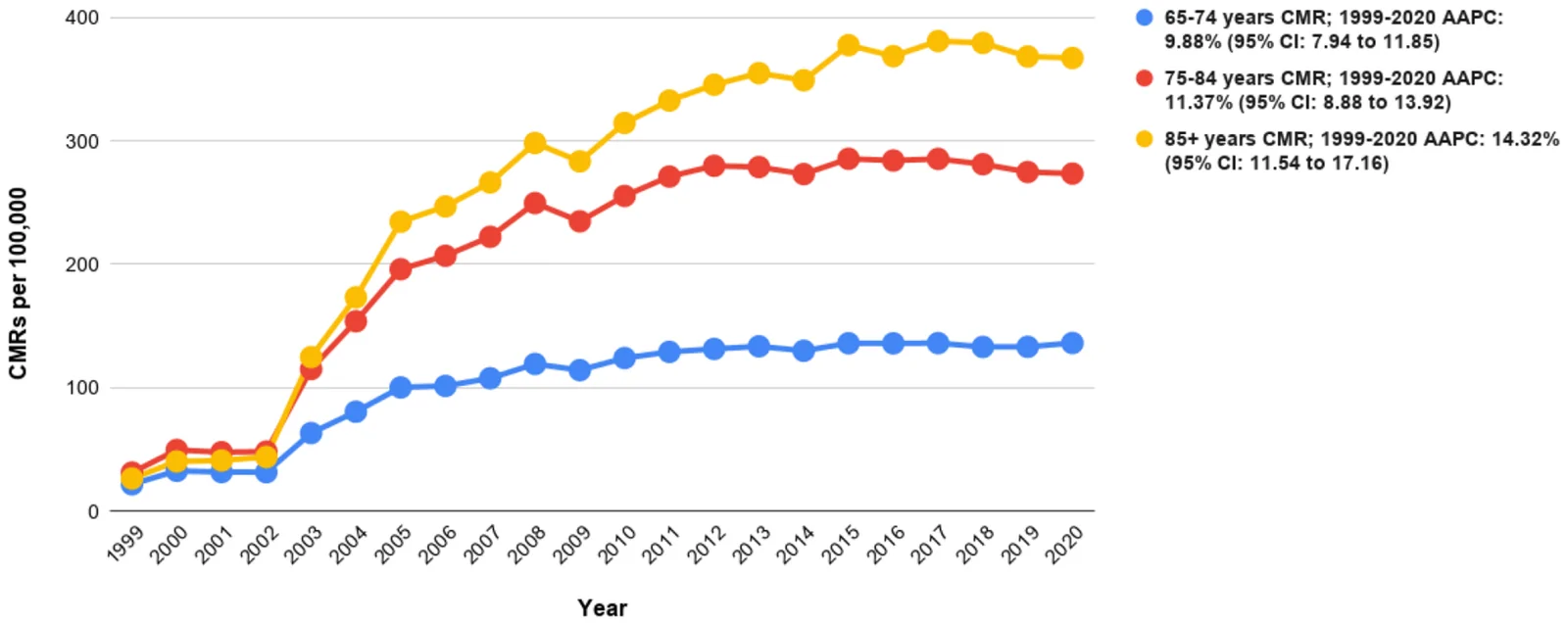
Chronic obstructive pulmonary disease and substance use disorder related AAMR per 100,000 by 10-year age group, United States, 1999 to 2020. AAMR = age-adjusted mortality rate, AAPC = average annual percent change, CI = confidence interval, CMR = crude mortality rate.

### 3.6. Urbanization stratification

From 1999 to 2020, 1,139,273 qualifying deaths occurred in metropolitan counties and 384,943 in nonmetropolitan counties (Table S1, Supplemental Digital Content 1). Despite fewer absolute deaths, nonmetropolitan counties had a higher pooled AAMR than metropolitan counties (232.82 vs 152.03/100,000). In metropolitan counties, mortality increased sharply from 1999 to 2005 (APC, 37.30%; 95% CI, 28.48–46.72), increased more gradually from 2005 to 2012 (APC, 4.30%; 95% CI, 0.75–7.98), and was stable from 2012 to 2020 (APC, −0.13%; 95% CI, −2.00 to 1.78), yielding an overall AAPC of 10.97% (95% CI, 8.63–13.37). In nonmetropolitan counties, mortality increased from 1999 to 2004 (APC, 41.60%; 95% CI, 28.99–55.43) and from 2004 to 2012 (APC, 7.92%; 95% CI, 4.93–11.01), followed by a statistically stable trend from 2012 to 2020 (APC, 1.02%; 95% CI, −0.79 to 2.87). The overall AAPC in nonmetropolitan counties was 12.27% (95% CI, 9.67–14.93) (Fig. 5, Table 1; Tables S6 and S7, Supplemental Digital Content 6).

**Figure 5.**
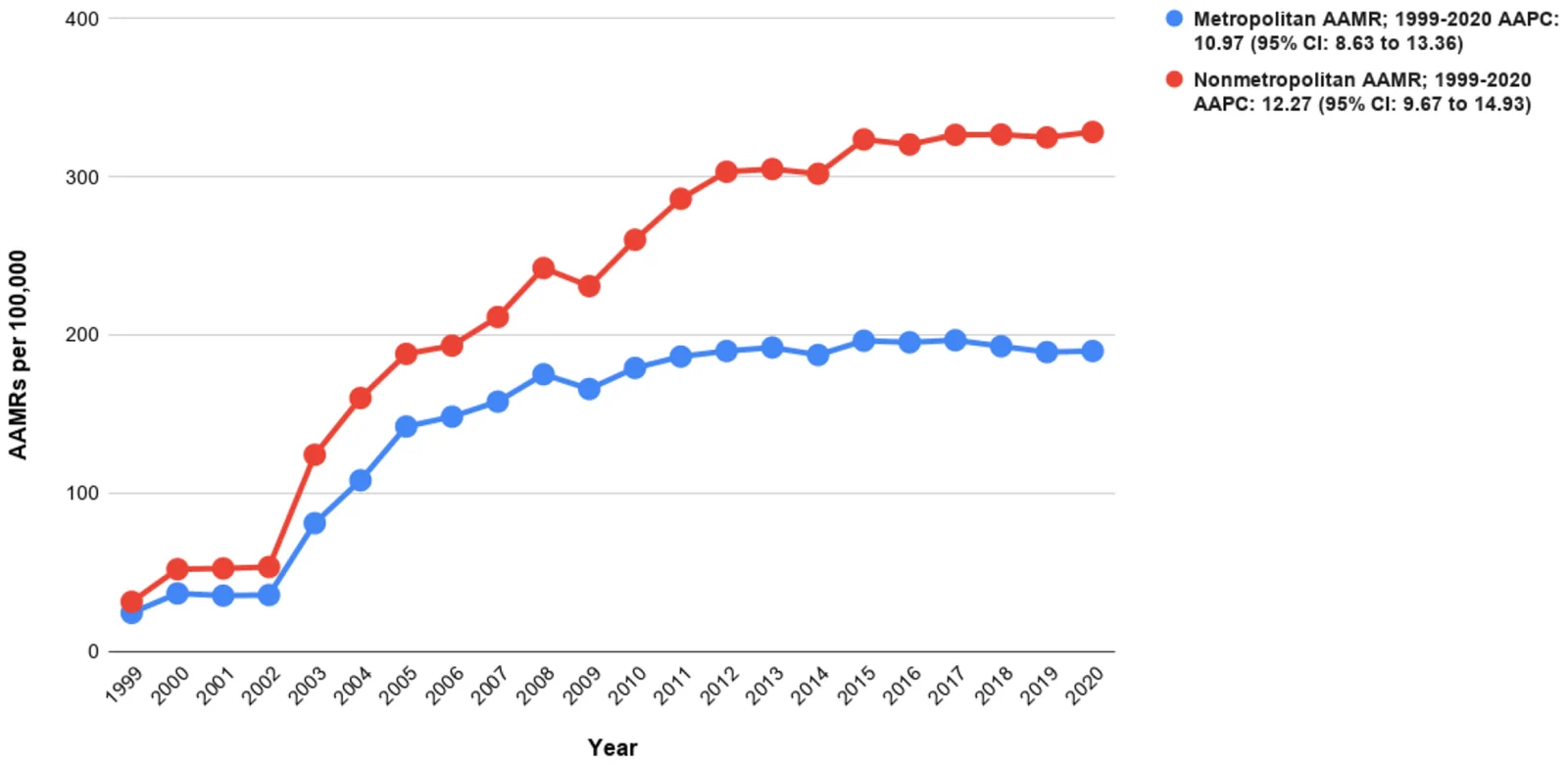
Chronic obstructive pulmonary disease and substance use disorder related AAMR per 100,000 by urbanization, United States, 1999 to 2020. AAMR = age-adjusted mortality rate, AAPC = average annual percent change, CI = confidence interval.

### 3.7. State stratification

State-level AAMRs for death records co-listing COPD and SUDs varied substantially across the United States. Across the pooled 1999 to 2020 period, the highest AAMR was observed in Vermont (409.3/100,000), followed by Wyoming (389.3), Oregon (371.3), Montana (368.5), and Idaho (363.7). The lowest pooled AAMRs were observed in California (32.0), Mississippi (52.3), Massachusetts (60.1), Alabama (61.4), and Virginia (74.1) (Fig. 6; Table S4, Supplemental Digital Content 7). These state-level estimates are descriptive and were not interpreted as causal effects of state residence.

**Figure 6.**
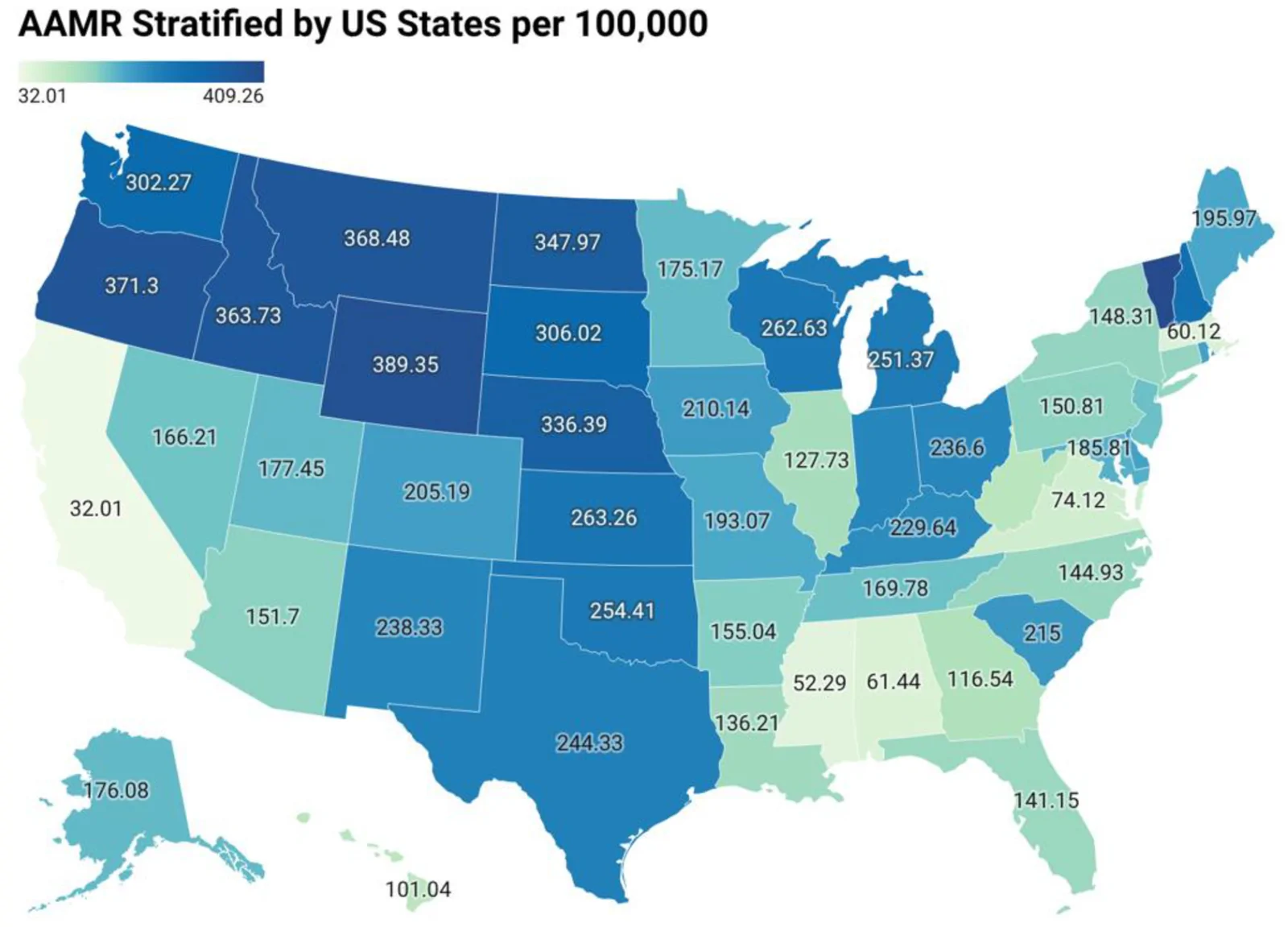
Chronic obstructive pulmonary disease and substance use disorder related AAMR per 100,000 by state, United States, 1999 to 2020. AAMR = age-adjusted mortality rate.

### 3.8. Substance-specific and sensitivity analysis

In the secondary substance-specific analysis, mortality burden and temporal patterns varied substantially across individual F10 to F19 categories. Tobacco/nicotine-related disorders (F17) accounted for the largest substance-specific mortality burden, with a pooled AAMR of 164.79/100,000, followed by alcohol-related disorders (F10; 3.39/100,000). The remaining substance-specific pooled AAMRs were substantially lower, including multiple/other psychoactive substance-related disorders (F19; 0.36/100,000) and opioid-related disorders (F11; 0.14/100,000). Several other categories had sparse or unreliable annual estimates and were therefore summarized descriptively (Table S9, Supplemental Digital Content 8).

Among categories with complete, reliable annual data, alcohol-related mortality initially declined from 1999 to 2010 (APC, −2.46%; 95% CI, −3.31 to −1.60) and subsequently increased from 2010 to 2020 (APC, 4.60%; 95% CI, 3.74–5.47), yielding an overall AAPC of 0.84% (95% CI, 0.28–1.41). Tobacco/nicotine-related mortality increased sharply from 1999 to 2005 (APC, 38.51%; 95% CI, 29.23–48.46), increased more slowly from 2005 to 2012 (APC, 4.87%; 95% CI, 1.26–8.62), and remained statistically stable from 2012 to 2020 (APC, 0.11%; 95% CI, −1.75 to 2.01), with an overall AAPC of 11.56% (95% CI, 9.13–14.04). Mortality involving multiple/other psychoactive substance-related disorders increased continuously over 1999–2020 (APC/AAPC, 10.61%; 95% CI, 9.67–11.56) (Table S10, Supplemental Digital Content 9).

In the sensitivity analysis excluding tobacco/nicotine-related disorders (F17), the overall AAMR increased from 3.61 /100,000 in 1999 to 5.91/100,000 in 2020. Mortality declined significantly from 1999 to 2009 (APC, −1.82%; 95% CI, −2.73 to −0.91), increased significantly from 2009 to 2017 (APC, 4.68%; 95% CI, 3.25–6.12), and increased more rapidly from 2017 to 2020 (APC, 9.55%; 95% CI, 5.36–13.90). The overall AAPC was 2.19% (95% CI, 1.37–3.02). This temporal pattern differed substantially from that observed in the primary F10 to F19 analysis, indicating that inclusion of tobacco/nicotine-related disorders materially influenced the aggregate mortality trend (Table S11A and B, Supplemental Digital Content 10).

## 4. Discussion

In this national descriptive analysis of U.S. adults aged ≥65 years, the overall F10 to F19 analysis showed a marked increase in mortality involving co-listed COPD and SUDs during the earlier study period, followed by stabilization through 2020. The overall AAMR increased from 25.5/100,000 in 1999 to a peak of 219.0 in 2017 and remained elevated at 213.2/100,000 in 2020. Important demographic and geographic differences were observed. Males had higher mortality than females; among racial and ethnic groups, the highest pooled AAMR was observed among NH American Indian or Alaska Native adults, followed closely by NH White adults. Age-specific mortality rates increased with advancing age, the Midwest had the highest pooled regional AAMR, and nonmetropolitan counties had higher AAMRs than metropolitan counties. Substantial state-level variation was also observed. These findings characterize population-level patterns among death records co-listing COPD and SUD diagnoses and should not be interpreted as evidence that SUDs caused COPD mortality or the observed temporal and subgroup differences. Importantly, this overall temporal pattern was not uniform across individual substance categories, which demonstrated heterogeneous trajectories in the substance-specific analyses.

The substance-specific analysis further demonstrated that the broad F10 to F19 grouping encompasses clinically heterogeneous conditions with markedly different mortality burdens and temporal patterns. Tobacco/nicotine-related disorders accounted for the predominant substance-specific burden, whereas alcohol-, opioid-, stimulant-, cocaine-, cannabinoid-, and other substance-related categories occurred at substantially lower rates. Temporal trajectories also differed across categories: alcohol-related mortality declined initially before increasing after 2010, tobacco/nicotine-related mortality rose markedly during the early study period before stabilizing after 2012, and mortality involving multiple/other psychoactive substance-related disorders increased throughout the study period. These findings reinforce that the aggregate F10 to F19 analysis should not be interpreted as representing a uniform pattern across individual substance-related diagnoses. The F17-excluded sensitivity analysis further highlighted the influence of tobacco/nicotine-related diagnoses on the aggregate F10 to F19 trend. Whereas the primary analysis showed a pronounced early increase followed by stabilization after 2012, exclusion of F17 revealed a substantially different temporal pattern, with mortality declining significantly through 2009, increasing thereafter, and rising most rapidly from 2017 to 2020. The overall AAPC in the F17-excluded analysis was 2.19% (95% CI, 1.37–3.02), substantially lower than that observed in the primary analysis. These findings indicate that inclusion of tobacco/nicotine-related disorders materially influenced the aggregate temporal trajectory and further support substance-specific interpretation of mortality patterns involving COPD.

The marked temporal increase during the earlier years of the study warrants cautious interpretation. Because both COPD and SUDs were selected through the CDC WONDER Multiple Cause of Death fields, with neither condition specified as the underlying cause of death, the analysis identifies deaths in which COPD and SUDs were co-listed among the multiple causes of death. Accordingly, it cannot determine which condition, if either, was the underlying cause of death or establish the temporal or causal relationship between them. Nevertheless, several clinically plausible pathways may contextualize their co-occurrence. Cigarette smoking remains the predominant preventable risk factor for COPD and is strongly associated with COPD morbidity and mortality.^[6,22,23]^ Other substance exposures may further compromise respiratory health through impaired respiratory function, treatment nonadherence, opioid-related ventilatory impairment, and greater health-care utilization.^[10,12,24–26]^ Among older adults, these risks may be compounded by reduced physiologic reserve, multimorbidity, polypharmacy, and increased susceptibility to adverse drug and substance effects.^[3,13,14]^ The subsequent stabilization in mortality may reflect changes in smoking and substance-use patterns, COPD recognition and management, health-care utilization, population characteristics, or death-certification practices; however, these potential explanations cannot be directly evaluated using CDC WONDER data.

The present study extends previous research by focusing specifically on older adults whose death certificates co-list COPD and SUDs. Prior U.S. studies have characterized COPD mortality according to sex, race/ethnicity, geography, and urbanization,^[7–9,19,27]^ while clinical and administrative studies have demonstrated substantial substance-use burden and increased health-care utilization among individuals with chronic obstructive lung disease.^[5,10–12]^ By examining long-term temporal trends together with demographic and geographic patterns among adults aged ≥65 years, our findings provide additional evidence that mortality involving co-listed COPD and SUDs is distributed unevenly across the older U.S. population.

Sex-specific differences were prominent, with males experiencing substantially higher AAMRs than females throughout the study period. Previous U.S. analyses have similarly demonstrated important sex differences in COPD mortality.^[7,9]^ This pattern may partly reflect historical differences in cumulative cigarette-smoking exposure, occupational inhalational exposures, comorbidity profiles, and patterns of substance use.^[9,12,27,28]^ However, these characteristics were not available at the individual level in CDC WONDER, and their contribution to the observed sex disparity cannot be quantified. The persistence of higher mortality among males nevertheless identifies an important population for COPD prevention, smoking cessation, substance-use assessment, and coordinated chronic-disease management.^[23,29]^

Mortality patterns also varied substantially across racial and ethnic groups. The highest pooled AAMR was observed among NH American Indian or Alaska Native adults, followed closely by NH White adults, whereas lower pooled rates were observed among NH Black or African American, Hispanic or Latino, and NH Asian or Pacific Islander adults. Previous studies have documented racial and sociodemographic variation in COPD mortality and tobacco exposure in the United States.^[9,27,28]^ The disparities observed here may reflect differences in smoking and substance-use patterns, occupational and environmental exposures, socioeconomic conditions, comorbidity burden, access to health care, and the recognition and certification of COPD and SUDs on death certificates. These findings should not be interpreted as reflecting intrinsic biological differences between racial or ethnic groups.^[27,30]^ Because these factors were not available at the individual level, their respective contributions cannot be determined from the present analysis.

Age was another important dimension of the observed mortality burden. Mortality increased across older age groups, with adults aged ≥85 years experiencing the highest rates. Advanced age is associated with declining physiologic reserve, increasing multimorbidity, and greater vulnerability to adverse COPD outcomes.^[3,13]^ Older adults may also be particularly susceptible to medication- and substance-related adverse effects, including opioid-related ventilatory impairment and complications associated with polypharmacy.^[14,25,26]^ Importantly, unhealthy substance use may be under-recognized in older populations, supporting continued clinical attention to substance-use assessment even at advanced ages.^[14,29]^

Marked geographic variation was also observed. The Midwest demonstrated the highest overall regional AAMR, while substantial variation was present across individual states. The greater burden observed in the Midwest may partly reflect the clustering of established COPD-related and substance-use risk factors in portions of the region. National surveillance has documented comparatively high tobacco-product use among adults living in the Midwest, as well as important socioeconomic disparities in tobacco exposure.^[28,31,32]^ Geographic and sociodemographic factors have also been associated with variation in COPD outcomes and mortality.^[2,7,27]^ Occupational and environmental exposures, socioeconomic disadvantage, health-care availability, and regional differences in substance-use patterns may additionally contribute; however, these characteristics were not directly measured in our analysis and should be considered possible contextual explanations rather than causal determinants.

Nonmetropolitan counties demonstrated higher AAMRs than metropolitan counties throughout the study period. This pattern is consistent with broader U.S. literature describing a rural respiratory-health disadvantage, including greater COPD prevalence and mortality in rural populations.^[19,33]^ Potential contextual factors include differences in tobacco exposure, occupational and environmental risks, socioeconomic conditions, health-care availability, and substance-use patterns. However, these factors were not directly measured in the present analysis and, therefore, cannot be considered causal explanations. The urbanization findings should instead be interpreted as descriptive geographic disparities warranting more detailed patient- and community-level investigation.

The present findings may have implications for surveillance and future research concerning co-occurring COPD and substance use in older adults. Substance-use assessment remains an important area for further investigation in this population, in whom unhealthy substance use may be under-recognized.^[14,29]^ However, because the present study was based on aggregate death-certificate data and did not evaluate screening, treatment, referral pathways, or other interventions, no conclusions can be drawn regarding the effectiveness of specific clinical or public-health strategies.^[23,34]^ The observed demographic and geographic patterns may instead help identify areas for further patient-level investigation, including research examining substance-use assessment, access to pulmonary and addiction care, treatment engagement, medication adherence, and other potentially modifiable factors,^[12,14,24,29,30,35,36]^ while treatment of alcohol and other SUDs should be individualized according to the substance involved, comorbidity burden, medication use, and patient preferences.^[14,34]^ Finally, the temporal increase observed in the overall co-listed COPD and SUD analysis should not be interpreted as evidence of a worsening biological interaction between the 2 conditions. Changes over time in smoking and substance-use prevalence, diagnostic recognition, health-care utilization, coding practices, death-certificate completion, and population characteristics may influence how frequently both conditions are recorded. These potential explanations cannot be directly evaluated using CDC WONDER data. Future studies using longitudinal clinical datasets with validated diagnoses and information on smoking exposure, specific substances, COPD severity, pulmonary function, comorbidities, socioeconomic conditions, health-care access, and treatment are needed to clarify the factors underlying the observed temporal and subgroup patterns and to evaluate potential causal pathways.

### 4.1. Strengths

This study has several strengths. CDC WONDER provides national coverage of mortality records from all 50 states and the District of Columbia over more than 2 decades, enabling consistent evaluation of temporal and geographic patterns. The use of standardized AAMRs and Joinpoint regression allowed changes in mortality trends to be summarized systematically. The analysis also evaluated multiple demographic and geographic strata and focused on adults aged ≥65 years, a clinically important population in whom COPD, multimorbidity, and substance-related risks may overlap.

### 4.2. Limitations

Several limitations should be considered. First, CDC WONDER relies on death-certificate data, and COPD or substance use disorder diagnoses may be under-recorded, miscoded, or documented differently across clinicians, institutions, states, and calendar years. Second, our case definition required both COPD and SUDs to be recorded in the Multiple Cause of Death fields, without restricting either condition to the underlying cause of death. Accordingly, this analysis identifies co-occurrence on death certificates but cannot determine which condition was the underlying cause of death, whether 1 contributed to the other, or the temporal or causal sequence between them. The findings should therefore be interpreted as mortality involving co-listed COPD and SUDs rather than mortality caused by SUDs among patients with COPD.

Third, although we performed secondary substance-specific analyses and an additional sensitivity analysis excluding tobacco/nicotine-related disorders (F17), the F10 to F19 grouping encompasses clinically heterogeneous disorders with substantially different prevalence, respiratory effects, and comorbidity patterns. Several substance-specific categories had sparse, suppressed, or unreliable annual estimates, limiting stable trend estimation and direct comparison across all substance types. In addition, substance-specific categories were not mutually exclusive because >1 substance-related diagnosis could be recorded on the same death certificate. Although exclusion of F17 reduced the direct influence of tobacco/nicotine-related diagnoses, CDC WONDER does not provide individual-level information on smoking history, intensity, duration, cessation status, specific substance dose, recency, severity, or polysubstance exposure; therefore, residual confounding related to tobacco and other substance exposures remains possible.

Fourth, other important potential confounders, including socioeconomic status, environmental and occupational exposures, COPD severity, pulmonary function, treatment adherence, comorbidities, health-care access, and insurance status, were unavailable, precluding multivariable adjustment. Fifth, the data are aggregate and cross-sectional at the mortality-record level rather than longitudinal patient-level observations; therefore, incidence, disease progression, repeated events, and individual treatment effects could not be assessed. Finally, changes over time in coding practices, diagnostic recognition, and death-certificate completion may have influenced the observed temporal trends. These limitations require cautious interpretation of the findings and preclude causal inference.

## 5. Conclusions

In the overall F10 to F19 analysis, mortality involving co-listed COPD and SUDs among U.S. adults aged ≥65 years increased markedly during the earlier study period before subsequently stabilizing. Substance-specific trajectories were heterogeneous and should not be assumed to follow the overall pattern. Substantial differences were observed across sex, race/ethnicity, age, census region, urbanization, and state. These findings identify population-level patterns that may help prioritize further surveillance and patient-level research. Because the study is descriptive and lacks individual-level confounder data, the results should not be interpreted as evidence that SUDs directly caused COPD mortality or the observed subgroup disparities.

## Acknowledgments

The authors acknowledge the use of ChatGPT (OpenAI) to assist with language editing and improvement of the manuscript’s readability. The authors were solely responsible for the study design, data analysis, interpretation of findings, and the final content of the manuscript.

## Author contributions

**Conceptualization:** Mirza Ammar Arshad.

**Data curation:** Urwah Noor, Muhammad Hassan.

**Formal analysis:** Urwah Noor, Mirza Ammar Arshad.

**Investigation:** Urwah Noor, Muhammad Shumas, Mirza Ammar Arshad.

**Project administration:** Mirza Ammar Arshad, Urwah Noor.

**Supervision:** Mirza Ammar Arshad.

**Visualization:** Muhammad Shumas, Amna Zafar.

**Writing – original draft:** Fatima Maqsood, Hassan Faraz Chaudhry, Amna Zafar, Menahil Fazal, Ravi Kumar Pandey, Rida Khan, Muhammad Hassan, Ashish Kumar, Abdullah Naeem, Usman Zia.

**Writing – review & editing:** Mirza Ammar Arshad, Urwah Noor, Rida Fatima, Yahye Ahmed Nor.






















