# Supplementary material for: National trends and demographic disparities in mortality involving co-listed chronic obstructive pulmonary disease and substance use disorders among U.S. adults aged ≥65 years: A population-based serial cross-sectional CDC WONDER analysis, 1999 to 2020
Source: Medicine (Baltimore). 2026 Nov 6;105(45):e51448. doi: 10.1097/MD.0000000000051448 (PMC13747554; doi:10.1097/MD.0000000000051448)
Supplement: Supplementary file 1 [file medi-105-e51448-s001.docx]

| **Variable** | **Deaths** | **% of total deaths** |
| --- | --- | --- |
| Overall | 1,524,216 | 100.00% |
| **Sex** | | |
| Male | 817,371 | 53.63% |
| Female | 706,845 | 46.37% |
| **Race/Ethnicity** | | |
| Non-Hispanic White | 1,394,789 | 91.51% |
| Non-Hispanic Black or African American | 75,333 | 4.94% |
| Non-Hispanic Asian or Pacific Islander | 9,088 | 0.60% |
| Non-Hispanic American Indian or Alaska Native | 8,433 | 0.55% |
| Hispanic or Latino | 33,054 | 2.17% |
| **Census region** | | |
| Northeast | 271,445 | 17.81% |
| Midwest | 448,713 | 29.44% |
| South | 539,930 | 35.42% |
| West | 264,128 | 17.33% |
| **Urbanization** |  |  |
| Metropolitan | 1,139,273 | 74.74% |
| Nonmetropolitan | 384,943 | 25.26% |
| **Age group** | | |
| 65–74 years | 555,060 | 36.42% |
| 75–84 years | 637,038 | 41.79% |
| ≥85 years | 332,118 | 21.79% |

**Supplementary Table 1.** Absolute number and percentage of deaths involving co-listed COPD and substance use disorders among U.S. adults aged ≥65 years, stratified by demographic characteristics, 1999–2020. Race/ethnicity categories shown may not sum to the overall number of deaths because records with other, unknown, or unavailable race/ethnicity classifications are not displayed.
