# Supplementary material for: National trends and demographic disparities in mortality involving co-listed chronic obstructive pulmonary disease and substance use disorders among U.S. adults aged ≥65 years: A population-based serial cross-sectional CDC WONDER analysis, 1999 to 2020
Source: Medicine (Baltimore). 2026 Nov 6;105(45):e51448. doi: 10.1097/MD.0000000000051448 (PMC13747554; doi:10.1097/MD.0000000000051448)
Supplement: Supplementary file 2 [file medi-105-e51448-s002.docx]

| **Year** | **Overall** | **Male** | **Female** |
| --- | --- | --- | --- |
| **1999** | 25.5 (25.0 - 26.1) | 35.83 (34.82– 36.84) | 18.98 (18.39– 19.58) |
| **2000** | 39.4 (38.8 - 40.1) | 54.05 (52.82– 55.28) | 30.08 (29.33– 30.83) |
| **2001** | 38.4 (37.7 - 39.0) | 52 (50.8– 53.2) | 29.49 (28.75– 30.23) |
| **2002** | 38.8 (38.2 - 39.5) | 51.75 (50.56– 52.94) | 30.55 (29.8– 31.30 |
| **2003** | 89.1 (88.1 - 90.0) | 120.13 (118.32– 121.940 | 69.3 (68.18– 70.42) |
| **2004** | 117.8 (116.7 - 118.9) | 158.65 (156.58– 160.72) | 91.99 (90.7– 93.28) |
| **2005** | 150.6 (149.3 - 151.8) | 200.7 (198.4– 203) | 118.4 (116.95– 119.85) |
| **2006** | 156.6 (155.3 - 157.9) | 208.68 (206.36– 211) | 122.73 (121.26– 124.2) |
| **2007** | 167.7 (166.4 - 169.0) | 221.22 (218.86– 223.59) | 132.75 (131.23– 134.27) |
| **2008** | 187.3 (186.0 - 188.7) | 244.35 (241.9– 246.8) | 149.8 (148.2– 151.41) |
| **2009** | 177.7 (176.3 - 179.0) | 230.18 (227.83– 232.54) | 142.61 (141.06– 144.17) |
| **2010** | 193.9 (192.5 - 195.3) | 250.07 (247.63– 252.5) | 156 (154.38– 157.62) |
| **2011** | 204.3 (202.9 - 205.7) | 263.65 (261.2– 266.11) | 163.73 (162.09– 165.37) |
| **2012** | 210.2 (208.8 - 211.6) | 270.44 (267.99– 272.89) | 168.88 (167.23– 170.52) |
| **2013** | 212.1 (210.7 - 213.5) | 269.82 (267.41– 272.23) | 172.14 (170.49– 173.78) |
| **2014** | 207.5 (206.2 - 208.8) | 263.38 (261.04– 265.72) | 168.05 (166.45– 169.66) |
| **2015** | 218.6 (217.2 - 220.0) | 274.46 (272.11– 276.82) | 178.77 (177.14– 180.4) |
| **2016** | 216.9 (215.6 - 218.3) | 274.25 (271.93– 276.57) | 175.85 (174.25– 177.45) |
| **2017** | 219.0 (217.7 - 220.3) | 274.4 (272.12– 276.68) | 178.84 (177.24– 180.43) |
| **2018** | 215.7 (214.4 - 217.0) | 270.33 (268.1– 272.55) | 175.65 (174.1– 177.21) |
| **2019** | 212.1 (210.9 - 213.4) | 265.67 (263.5– 267.84) | 172.46 (170.95– 173.98) |
| **2020** | 213.2 (211.9 - 214.4) | 270.76 (268.61– 272.92) | 169.98 (168.5– 171.46) |
| **Total** | 166.6 (166.3 - 166.9) | 216.98 (216.51– 217.46) | 132.54 (132.23– 132.85) |

**Supplementary Table 2:** AAMRs per 100,000 overall and by sex for deaths involving co-listed COPD and substance use disorders among U.S. adults aged ≥65 years, 1999–2020.
