# Supplementary material for: National trends and demographic disparities in mortality involving co-listed chronic obstructive pulmonary disease and substance use disorders among U.S. adults aged ≥65 years: A population-based serial cross-sectional CDC WONDER analysis, 1999 to 2020
Source: Medicine (Baltimore). 2026 Nov 6;105(45):e51448. doi: 10.1097/MD.0000000000051448 (PMC13747554; doi:10.1097/MD.0000000000051448)
Supplement: Supplementary file 3 [file medi-105-e51448-s003.docx]

| **Year** | **NH American Indian or Alaska Native** | **NH Asian or Pacific Islander** | **NH Black or African American** | **NH White** | **Hispanic or Latino** |
| --- | --- | --- | --- | --- | --- |
| 1999 | 37.88 | 6.29 | 17.36 | 27.45 | 12.28 |
| 2000 | 59.08 | 8.04 | 22.19 | 43.14 | 16.25 |
| 2001 | 50.29 | 7.16 | 21.72 | 42.21 | 15.05 |
| 2002 | 54.06 | 9.12 | 22.98 | 42.7 | 14.56 |
| 2003 | 87.6 | 12.42 | 46.03 | 98.54 | 43.86 |
| 2004 | 177.56 | 17.41 | 58.37 | 131.5 | 43.62 |
| 2005 | 189.56 | 19.05 | 78.77 | 168.27 | 58.46 |
| 2006 | 212.11 | 31.18 | 81.71 | 174.97 | 59.49 |
| 2007 | 212.72 | 32.71 | 90.02 | 188.38 | 57.16 |
| 2008 | 190.27 | 33.92 | 100.43 | 211.57 | 60.5 |
| 2009 | 179.77 | 32.48 | 92.19 | 201.58 | 55.8 |
| 2010 | 217.62 | 33.8 | 104.68 | 220.14 | 61.62 |
| 2011 | 213.85 | 33.31 | 108.61 | 234.05 | 58.27 |
| 2012 | 199.36 | 30.85 | 114.4 | 241.18 | 62.35 |
| 2013 | 208.28 | 32.46 | 117.6 | 243.83 | 65.07 |
| 2014 | 232.38 | 30.7 | 117.87 | 239.1 | 62.26 |
| 2015 | 220.78 | 30.84 | 125.23 | 253.05 | 64.95 |
| 2016 | 239.19 | 34.16 | 130.66 | 251.41 | 62.41 |
| 2017 | 249.41 | 32.43 | 131.05 | 255.19 | 60.3 |
| 2018 | 234.87 | 31.15 | 132.7 | 251.52 | 61.65 |
| 2019 | 221.11 | 29.76 | 129.58 | 248.75 | 58.32 |
| 2020 | 215.1 | 33.85 | 143.35 | 248.24 | 66.92 |
| **Overall** | **191.99** | **28.41** | **97.04** | **188.68** | **55.35** |

**Supplementary Table 3.** Race/ethnicity-stratified AAMRs per 100,000 among U.S. adults aged ≥65 years with co-listed chronic obstructive pulmonary disease and substance use disorders, 1999–2020.
