# Supplementary material for: National trends and demographic disparities in mortality involving co-listed chronic obstructive pulmonary disease and substance use disorders among U.S. adults aged ≥65 years: A population-based serial cross-sectional CDC WONDER analysis, 1999 to 2020
Source: Medicine (Baltimore). 2026 Nov 6;105(45):e51448. doi: 10.1097/MD.0000000000051448 (PMC13747554; doi:10.1097/MD.0000000000051448)
Supplement: Supplementary file 4 [file medi-105-e51448-s004.docx]

| **Age Adjusted Rate/100,000 (95% CI)** | | | | |
| --- | --- | --- | --- | --- |
| **Year** | **Northeast** | **Midwest** | **South** | **West** |
| **1999** | 13.8 (13 –14.7) | 23.5 (22.5 – 24.6) | 24.7 (23.8 – 25.6) | 42.3 (40.7 – 43.8) |
| **2000** | 23.7 (22.6 – 24.8) | 38.9 (37.5 – 40.2) | 40.3 (39.2 – 41.4) | 55.4 (53.7 – 57.2) |
| **2001** | 23.9 (22.8 – 25.1) | 38.4 (37 – 39.7) | 38.7 (37.6 – 39.8) | 53 (51.3 – 54.7) |
| **2002** | 24.7 (23.6 – 25.9) | 39.5 (38.2 – 40.9) | 38.7 (37.7 – 39.8) | 53 (51.3 – 54.7) |
| **2003** | 77 (75 – 79) ­ | 72.9 (71.1– 74.7) | 105 (103.3 – 106.8) | 92.3 (90.1 – 94.5) |
| **2004** | 124 ­­­(121.5 – 126.6) | 117.1 (114.8 – 119.5) | 108.1 (106.3 – 109.9) | 128.5 (125.9 –131.1) |
| **2005** | 141.3 (138.6 – 144) | 145.5 (142.9 –148) | 168.2 (166 – 170.4) | 134.8 (132.1 – 137.4) |
| **2006** | 188.4 (185.3 – 191.5) | 141.6 (139.1 – 144.2) | 157.3 (155.2 – 159.4) | 140.1 (137.4 – 142.7) |
| **2007** | 191.7 (188.6 – 194.8) | 192.5 (189.6 – 195.4) | 155.8 (153.7–157.9) | 137.1 (134.5 – 139.6) |
| **2008** | 194.2 (191.1 – 197.3) | 260.1 (256.7 – 263.4) | 161.5 (159.4 –163.6) | 145.3 (142.7 – 148) |
| **2009** | 186.8 (183.8 – 189.9) | 244.7 (241.4 – 247.9 | 154.2 (152.2 – 156.3) | 136.5 (133.9 – 139) |
| **2010** | 182.4 (179.4 – 185.4) | 272.4 (269 – 275.8) | 172.6 (170.4 –174.7) | 156.9 (154.2 –159.6) |
| **2011** | 184 (181 – 187) | 313.6 (310 – 317.3) | 174.4 (172.3 – 176.5) | 158.3 (155.7 –161) |
| **2012** | 191.8 (188.7 – 194.8) | 313.6 (310 – 317.2) | 185.6 (183.4 – 187.7) | 161.1 (158.4 –163.7) |
| **2013** | 190.6 (187.7 – 193.6) | 313.6 (310 – 317.2) | 190 (187.8 –192.1) | 164.5 (161.9 – 167.2) |
| **2014** | 181.5 (178.6 – 184.4) | 305.6 (302.1 – 309.1) | 192.8 (190.7 – 194.9) | 155.4 (152.9 – 157.9) |
| **2015** | 198 (195 – 201) | 302.1 (298.7 – 305.5) | 210.3 (208.1 – 212.5) | 166.5 (163.9 – 169) |
| **2016** | 189.1 (186.2 – 192) | 302.2 (298.8 – 305.6) | 210.6 (208.4 – 212.7) | 167.3 (164.8 – 169.8) |
| **2017** | 187.9 (185 – 190.7) | 302.8 (299.5 – 306.2) | 218.1 (215.9 – 220.2) | 164.2 (161.7 –166.6) |
| **2018** | 183.7 (180.9 – 186.5) | 293.2 (290 – 296.5) | 220.5 (218.4 – 222.6) | 158.7 (156.4 –161.1) |
| **2019** | 180.3 (177.6 – 183.1) | 288.4 (285.2 –291.6) | 217 (214.9 – 219) | 157 (154.7 –159.3) |
| **2020** | 177.3 (174.7 – 180) | 296.4 (293.2 –299.6) | 216.9 (214.9 – 218.9) | 156.6 (154.4 –158.9) |
| **Total** | 150.1 (149.5 – 150.7) | 217.7 (217.1 – 218.3) | 161.4 (160.9 –161.8) | 136.4 (135.8 –136.9) |

**Supplementary Table 5:** AAMRs per 100,000 by U.S. Census region for deaths involving co-listed COPD and substance use disorders among adults aged ≥65 years, 1999–2020.
