# Supplementary material for: National trends and demographic disparities in mortality involving co-listed chronic obstructive pulmonary disease and substance use disorders among U.S. adults aged ≥65 years: A population-based serial cross-sectional CDC WONDER analysis, 1999 to 2020
Source: Medicine (Baltimore). 2026 Nov 6;105(45):e51448. doi: 10.1097/MD.0000000000051448 (PMC13747554; doi:10.1097/MD.0000000000051448)
Supplement: Supplementary file 5 [file medi-105-e51448-s005.docx]

| **Year** | **65–74 years** | **75–84 years** | **≥85 years** |
| --- | --- | --- | --- |
| 1999 | 21.60 (20.93–22.27) | 31.14 (30.15–32.13) | 26.31 (24.75–27.87) |
| 2000 | 32.49 (31.67–33.32) | 49.44 (48.20–50.68) | 40.05 (38.15–41.96) |
| 2001 | 31.49 (30.68–32.31) | 47.63 (46.42–48.83) | 40.88 (38.97–42.79) |
| 2002 | 31.44 (30.63–32.25) | 48.12 (46.91–49.32) | 43.65 (41.69–45.61) |
| 2003 | 63.13 (61.99–64.28) | 115.05 (113.20–116.90) | 124.67 (121.40–127.95) |
| 2004 | 80.40 (79.12–81.69) | 153.71 (151.58–155.84) | 173.08 (169.26–176.90) |
| 2005 | 100.15 (98.72–101.58) | 195.90 (193.50–198.30) | 234.40 (230.02–238.78) |
| 2006 | 101.25 (99.83–102.68) | 206.83 (204.37–209.30) | 246.78 (242.36–251.19) |
| 2007 | 107.54 (106.09–108.99) | 222.21 (219.66–224.77) | 266.21 (261.71–270.72) |
| 2008 | 119.15 (117.65–120.64) | 249.54 (246.83–252.25) | 298.18 (293.49–302.88) |
| 2009 | 114.10 (112.66–115.54) | 234.69 (232.06–237.32) | 283.29 (278.79–287.79) |
| 2010 | 123.90 (122.42–125.38) | **255.25 (252.51–257.99)** | 314.36 (309.67–319.05) |
| 2011 | 128.88 (127.40–130.37) | 271.02 (268.21–273.83) | 332.55 (327.83–337.27) |
| 2012 | 131.30 (129.85–132.75) | 279.66 (276.81–282.50) | 345.44 (340.69–350.18) |
| 2013 | 133.42 (131.99–134.84) | 278.67 (275.85–281.49) | 354.77 (350.02–359.52) |
| 2014 | 129.80 (128.43–131.17) | 272.95 (270.18–275.72) | 349.01 (344.35–353.68) |
| 2015 | 135.88 (134.50–137.25) | 285.38 (282.57–288.19) | 377.47 (372.67–382.27) |
| 2016 | 135.82 (134.47–137.17) | 284.02 (281.25–286.79) | 368.62 (363.91–373.33) |
| 2017 | 135.95 (134.62–137.28) | 285.25 (282.52–287.98) | 380.82 (376.06–385.58) |
| 2018 | 132.91 (131.61–134.20) | 281.02 (278.38–283.67) | 379.26 (374.55–383.98) |
| 2019 | 133.01 (131.73–134.28) | 274.74 (272.17–277.31) | 368.31 (363.69–372.94) |
| 2020 | 136.08 (134.81–137.35) | 273.35 (270.83–275.88) | 367.08 (362.48–371.69) |
| **Overall** | **108.74 (108.45–109.02)** | **213.41 (212.89–213.93)** | **277.89 (276.95–278.84)** |

**Supplementary Table 8:** Age-group–stratified crude mortality rates per 100,000 among U.S. adults aged ≥65 years with co-listed COPD and substance use disorders, 1999–2020
