# Supplementary material for: National trends and demographic disparities in mortality involving co-listed chronic obstructive pulmonary disease and substance use disorders among U.S. adults aged ≥65 years: A population-based serial cross-sectional CDC WONDER analysis, 1999 to 2020
Source: Medicine (Baltimore). 2026 Nov 6;105(45):e51448. doi: 10.1097/MD.0000000000051448 (PMC13747554; doi:10.1097/MD.0000000000051448)
Supplement: Supplementary file 6 [file medi-105-e51448-s006.docx]

| **Variable** | **Interval** | **APC (95% CI)** |
| --- | --- | --- |
| **Overall** | 1999–2005 | 36.52 (27.74 to 45.90) |
|  | 2005–2012 | 4.86 (1.30 to 8.56) |
|  | 2012–2020 | 0.15 (−1.70 to 2.03) |
| **Sex** | | |
| Female | 1999–2005 | 37.33 (28.48 to 46.79) |
|  | 2005–2012 | 5.34 (1.76 to 9.04) |
|  | 2012–2020 | 0.13 (−1.71 to 2.01) |
| Male | 1999–2005 | 38.92 (29.05 to 49.55) |
|  | 2005–2020 | 1.72 (0.84 to 2.62) |
| **Race/Ethnicity** | | |
| NH American Indian or Alaska Native | 1999–2005 | 34.92 (22.27 to 48.88) |
|  | 2005–2020 | 1.12 (−0.06 to 2.31) |
| NH Asian or Pacific Islander | 1999–2007 | 25.63 (20.39 to 31.10) |
|  | 2007–2020 | −0.42 (−1.30 to 0.47) |
| NH Black or African American | 1999–2006 | 28.74 (23.01 to 34.74) |
|  | 2006–2020 | 3.25 (2.47 to 4.04) |
| NH White | 1999–2005 | 37.06 (28.18 to 46.55) |
|  | 2005–2012 | 5.28 (1.68 to 9.01) |
|  | 2012–2020 | 0.40 (−1.47 to 2.30) |
| Hispanic or Latino | 1999–2005 | 32.37 (20.80 to 45.05) |
|  | 2005–2020 | 0.49 (−0.53 to 1.53) |
| **Census region** | | |
| Northeast | 1999–2006 | 43.51 (34.17 to 53.49) |
|  | 2006–2020 | −0.61 (−1.64 to 0.42) |
| Midwest | 1999–2008 | 27.82 (22.81 to 33.04) |
|  | 2008–2012 | 5.56 (−4.85 to 17.11) |
|  | 2012–2020 | −0.96 (−2.95 to 1.07) |
| South | 1999–2005 | 35.58 (25.44 to 46.53) |
|  | 2005–2020 | 2.64 (1.71 to 3.58) |
| West | 1999–2005 | 23.12 (16.27 to 30.37) |
|  | 2005–2015 | 2.00 (0.04 to 4.01) |
|  | 2015–2020 | −1.36 (−5.28 to 2.72) |
| **Urbanization** | | |
| Metropolitan | 1999–2005 | 37.30 (28.48 to 46.72) |
|  | 2005–2012 | 4.30 (0.75 to 7.98) |
|  | 2012–2020 | −0.13 (−2.00 to 1.78) |
| Nonmetropolitan | 1999–2004 | 41.60 (28.99 to 55.43) |
|  | 2004–2012 | 7.92 (4.93 to 11.01) |
|  | 2012–2020 | 1.02 (−0.79 to 2.87) |
| **Age group** | | |
| 65–74 years | 1999–2005 | 32.89 (24.67 to 41.65) |
|  | 2005–2020 | 1.84 (1.02 to 2.67) |
| 75–84 years | 1999–2005 | 38.21 (28.85 to 48.25) |
|  | 2005–2012 | 4.96 (1.18 to 8.89) |
|  | 2012–2020 | −0.22 (−2.26 to 1.87) |
| ≥85 years | 1999–2005 | 48.42 (36.61 to 61.25) |
|  | 2005–2013 | 5.20 (2.39 to 8.09) |
|  | 2013–2020 | 0.51 (−1.71 to 2.77) |

**Supplementary Table 6.** Annual percent change in mortality rates among U.S. adults aged ≥65 years with co-listed COPD and substance use disorders, 1999–2020. APC values are annual percent changes estimated using Joinpoint regression; 95% confidence intervals excluding zero indicate statistical significance.

**Abbreviations:** APC, annual percent change; CI, confidence interval; NH, non-Hispanic.
