# Supplementary material for: National trends and demographic disparities in mortality involving co-listed chronic obstructive pulmonary disease and substance use disorders among U.S. adults aged ≥65 years: A population-based serial cross-sectional CDC WONDER analysis, 1999 to 2020
Source: Medicine (Baltimore). 2026 Nov 6;105(45):e51448. doi: 10.1097/MD.0000000000051448 (PMC13747554; doi:10.1097/MD.0000000000051448)
Supplement: Supplementary file 7 [file medi-105-e51448-s007.docx]

| **State** | **Age Adjusted Rate** |
| --- | --- |
| Alabama | 61.44 |
| Alaska | 176.08 |
| Arizona | 151.7 |
| Arkansas | 155.04 |
| California | 32.01 |
| Colorado | 205.19 |
| Connecticut | 147.86 |
| Delaware | 202.33 |
| District of Columbia | 75.58 |
| Florida | 141.15 |
| Georgia | 116.54 |
| Hawaii | 101.04 |
| Idaho | 363.73 |
| Illinois | 127.73 |
| Indiana | 235.88 |
| Iowa | 210.14 |
| Kansas | 263.26 |
| Kentucky | 229.64 |
| Louisiana | 136.21 |
| Maine | 195.97 |
| Maryland | 185.81 |
| Massachusetts | 60.12 |
| Michigan | 251.37 |
| Minnesota | 175.17 |
| Mississippi | 52.29 |
| Missouri | 193.07 |
| Montana | 368.48 |
| Nebraska | 336.39 |
| Nevada | 166.21 |
| New Hampshire | 284.04 |
| New Jersey | 169.67 |
| New Mexico | 238.33 |
| New York | 148.31 |
| North Carolina | 144.93 |
| North Dakota | 347.97 |
| Ohio | 236.6 |
| Oklahoma | 254.41 |
| Oregon | 371.3 |
| Pennsylvania | 150.81 |
| Rhode Island | 202.4 |
| South Carolina | 215 |
| South Dakota | 306.02 |
| Tennessee | 169.78 |
| Texas | 244.33 |
| Utah | 177.45 |
| Vermont | 409.26 |
| Virginia | 74.12 |
| Washington | 302.27 |
| West Virginia | 102.36 |
| Wisconsin | 262.63 |
| Wyoming | 389.35 |

**Supplementary Table 4:** State-stratified AAMRs per 100,000 for deaths involving co-listed COPD and substance use disorders among U.S. adults aged ≥65 years, 1999–2020.
