# Supplementary material for: National trends and demographic disparities in mortality involving co-listed chronic obstructive pulmonary disease and substance use disorders among U.S. adults aged ≥65 years: A population-based serial cross-sectional CDC WONDER analysis, 1999 to 2020
Source: Medicine (Baltimore). 2026 Nov 6;105(45):e51448. doi: 10.1097/MD.0000000000051448 (PMC13747554; doi:10.1097/MD.0000000000051448)
Supplement: Supplementary file 8 [file medi-105-e51448-s008.docx]

| **ICD-10** | **Substance category** | **Year** | **Deaths** | **Population** | **Crude rate** | **AAMR** | **AAMR SE** |
| --- | --- | --- | --- | --- | --- | --- | --- |
| F10 | Alcohol-related disorders | 1999 | 1,220 | 34,797,841 | 3.51 | 3.52 | 0.1 |
| F10 | Alcohol-related disorders | 2000 | 1,263 | 34,991,753 | 3.61 | 3.63 | 0.1 |
| F10 | Alcohol-related disorders | 2001 | 1,201 | 35,290,291 | 3.4 | 3.42 | 0.1 |
| F10 | Alcohol-related disorders | 2002 | 1,180 | 35,522,207 | 3.32 | 3.32 | 0.1 |
| F10 | Alcohol-related disorders | 2003 | 1,185 | 35,863,529 | 3.3 | 3.32 | 0.1 |
| F10 | Alcohol-related disorders | 2004 | 1,120 | 36,203,319 | 3.09 | 3.06 | 0.09 |
| F10 | Alcohol-related disorders | 2005 | 1,188 | 36,649,798 | 3.24 | 3.24 | 0.09 |
| F10 | Alcohol-related disorders | 2006 | 1,162 | 37,164,107 | 3.13 | 3.13 | 0.09 |
| F10 | Alcohol-related disorders | 2007 | 1,042 | 37,825,711 | 2.75 | 2.76 | 0.09 |
| F10 | Alcohol-related disorders | 2008 | 1,093 | 38,777,621 | 2.82 | 2.86 | 0.09 |
| F10 | Alcohol-related disorders | 2009 | 1,109 | 39,623,175 | 2.8 | 2.81 | 0.08 |
| F10 | Alcohol-related disorders | 2010 | 1,173 | 40,267,984 | 2.91 | 2.95 | 0.09 |
| F10 | Alcohol-related disorders | 2011 | 1,221 | 41,394,141 | 2.95 | 2.93 | 0.08 |
| F10 | Alcohol-related disorders | 2012 | 1,239 | 43,145,356 | 2.87 | 2.85 | 0.08 |
| F10 | Alcohol-related disorders | 2013 | 1,446 | 44,704,074 | 3.23 | 3.2 | 0.08 |
| F10 | Alcohol-related disorders | 2014 | 1,534 | 46,243,211 | 3.32 | 3.29 | 0.08 |
| F10 | Alcohol-related disorders | 2015 | 1,652 | 47,760,852 | 3.46 | 3.43 | 0.09 |
| F10 | Alcohol-related disorders | 2016 | 1,782 | 49,244,195 | 3.62 | 3.54 | 0.08 |
| F10 | Alcohol-related disorders | 2017 | 1,854 | 50,858,679 | 3.65 | 3.6 | 0.08 |
| F10 | Alcohol-related disorders | 2018 | 2,075 | 52,431,193 | 3.96 | 3.88 | 0.09 |
| F10 | Alcohol-related disorders | 2019 | 2,157 | 54,058,263 | 3.99 | 3.92 | 0.09 |
| F10 | Alcohol-related disorders | 2020 | 2,669 | 55,659,365 | 4.8 | 4.62 | 0.09 |
| F11 | Opioid-related disorders | 2004 | 13 | 36,203,319 | Unreliable | Unreliable | 0 |
| F11 | Opioid-related disorders | 2007 | 12 | 37,825,711 | Unreliable | Unreliable | 0.02 |
| F11 | Opioid-related disorders | 2008 | 11 | 38,777,621 | Unreliable | Unreliable | 0 |
| F11 | Opioid-related disorders | 2009 | 21 | 39,623,175 | 0.05 | 0.05 | 0.01 |
| F11 | Opioid-related disorders | 2011 | 30 | 41,394,141 | 0.07 | 0.06 | 0.01 |
| F11 | Opioid-related disorders | 2012 | 29 | 43,145,356 | 0.07 | 0.05 | 0.01 |
| F11 | Opioid-related disorders | 2013 | 39 | 44,704,074 | 0.09 | 0.09 | 0.02 |
| F11 | Opioid-related disorders | 2014 | 65 | 46,243,211 | 0.14 | 0.15 | 0.02 |
| F11 | Opioid-related disorders | 2015 | 71 | 47,760,852 | 0.15 | 0.15 | 0.02 |
| F11 | Opioid-related disorders | 2016 | 81 | 49,244,195 | 0.16 | 0.14 | 0.02 |
| F11 | Opioid-related disorders | 2017 | 134 | 50,858,679 | 0.26 | 0.24 | 0.02 |
| F11 | Opioid-related disorders | 2018 | 169 | 52,431,193 | 0.32 | 0.34 | 0.03 |
| F11 | Opioid-related disorders | 2019 | 175 | 54,058,263 | 0.32 | 0.29 | 0.02 |
| F11 | Opioid-related disorders | 2020 | 220 | 55,659,365 | 0.4 | 0.38 | 0.03 |
| F12 | Cannabinoid-related disorders | 2016 | 10 | 49,244,195 | Unreliable | Unreliable | 0 |
| F12 | Cannabinoid-related disorders | 2017 | 18 | 50,858,679 | Unreliable | Unreliable | 0.01 |
| F12 | Cannabinoid-related disorders | 2018 | 17 | 52,431,193 | Unreliable | Unreliable | 0 |
| F12 | Cannabinoid-related disorders | 2019 | 27 | 54,058,263 | 0.05 | 0.05 | 0.01 |
| F12 | Cannabinoid-related disorders | 2020 | 20 | 55,659,365 | 0.04 | 0.05 | 0.01 |
| F13 | Sedative/hypnotic-related disorders | 2014 | 11 | 46,243,211 | Unreliable | Unreliable | 0 |
| F13 | Sedative/hypnotic-related disorders | 2017 | 16 | 50,858,679 | Unreliable | Unreliable | 0 |
| F13 | Sedative/hypnotic-related disorders | 2018 | 23 | 52,431,193 | 0.04 | 0 | 0 |
| F13 | Sedative/hypnotic-related disorders | 2019 | 16 | 54,058,263 | Unreliable | Unreliable | 0 |
| F13 | Sedative/hypnotic-related disorders | 2020 | 20 | 55,659,365 | 0.04 | 0 | 0 |
| F14 | Cocaine-related disorders | 2006 | 14 | 37,164,107 | Unreliable | Unreliable | 0.01 |
| F14 | Cocaine-related disorders | 2007 | 11 | 37,825,711 | Unreliable | Unreliable | 0.02 |
| F14 | Cocaine-related disorders | 2009 | 15 | 39,623,175 | Unreliable | Unreliable | 0 |
| F14 | Cocaine-related disorders | 2010 | 12 | 40,267,984 | Unreliable | Unreliable | 0 |
| F14 | Cocaine-related disorders | 2011 | 19 | 41,394,141 | Unreliable | Unreliable | 0.01 |
| F14 | Cocaine-related disorders | 2012 | 14 | 43,145,356 | Unreliable | Unreliable | 0 |
| F14 | Cocaine-related disorders | 2013 | 24 | 44,704,074 | 0.05 | 0.05 | 0.01 |
| F14 | Cocaine-related disorders | 2014 | 18 | 46,243,211 | Unreliable | Unreliable | 0.01 |
| F14 | Cocaine-related disorders | 2015 | 36 | 47,760,852 | 0.08 | 0.05 | 0.01 |
| F14 | Cocaine-related disorders | 2016 | 36 | 49,244,195 | 0.07 | 0.05 | 0.01 |
| F14 | Cocaine-related disorders | 2017 | 51 | 50,858,679 | 0.1 | 0.1 | 0.01 |
| F14 | Cocaine-related disorders | 2018 | 64 | 52,431,193 | 0.12 | 0.1 | 0.01 |
| F14 | Cocaine-related disorders | 2019 | 82 | 54,058,263 | 0.15 | 0.16 | 0.02 |
| F14 | Cocaine-related disorders | 2020 | 94 | 55,659,365 | 0.17 | 0.19 | 0.02 |
| F15 | Other stimulant-related disorders | 2014 | 23 | 46,243,211 | 0.05 | 0.05 | 0.01 |
| F15 | Other stimulant-related disorders | 2015 | 18 | 47,760,852 | Unreliable | Unreliable | 0.01 |
| F15 | Other stimulant-related disorders | 2016 | 20 | 49,244,195 | 0.04 | 0.05 | 0.01 |
| F15 | Other stimulant-related disorders | 2017 | 48 | 50,858,679 | 0.09 | 0.05 | 0.01 |
| F15 | Other stimulant-related disorders | 2018 | 55 | 52,431,193 | 0.1 | 0.1 | 0.01 |
| F15 | Other stimulant-related disorders | 2019 | 80 | 54,058,263 | 0.15 | 0.14 | 0.02 |
| F15 | Other stimulant-related disorders | 2020 | 107 | 55,659,365 | 0.19 | 0.16 | 0.02 |
| F17 | Tobacco/nicotine-related disorders | 1999 | 7,844 | 34,797,841 | 22.54 | 22.6 | 0.26 |
| F17 | Tobacco/nicotine-related disorders | 2000 | 12,813 | 34,991,753 | 36.62 | 36.68 | 0.32 |
| F17 | Tobacco/nicotine-related disorders | 2001 | 12,605 | 35,290,291 | 35.72 | 35.69 | 0.32 |
| F17 | Tobacco/nicotine-related disorders | 2002 | 12,912 | 35,522,207 | 36.35 | 36.26 | 0.32 |
| F17 | Tobacco/nicotine-related disorders | 2003 | 31,294 | 35,863,529 | 87.26 | 86.87 | 0.49 |
| F17 | Tobacco/nicotine-related disorders | 2004 | 42,162 | 36,203,319 | 116.46 | 115.92 | 0.56 |
| F17 | Tobacco/nicotine-related disorders | 2005 | 54,834 | 36,649,798 | 149.62 | 148.71 | 0.64 |
| F17 | Tobacco/nicotine-related disorders | 2006 | 57,911 | 37,164,107 | 155.83 | 154.88 | 0.64 |
| F17 | Tobacco/nicotine-related disorders | 2007 | 63,127 | 37,825,711 | 166.89 | 166.21 | 0.66 |
| F17 | Tobacco/nicotine-related disorders | 2008 | 72,021 | 38,777,621 | 185.73 | 185.98 | 0.69 |
| F17 | Tobacco/nicotine-related disorders | 2009 | 69,420 | 39,623,175 | 175.2 | 176.2 | 0.67 |
| F17 | Tobacco/nicotine-related disorders | 2010 | 76,907 | 40,267,984 | 190.99 | 192.35 | 0.7 |
| F17 | Tobacco/nicotine-related disorders | 2011 | 83,196 | 41,394,141 | 200.98 | 202.95 | 0.71 |
| F17 | Tobacco/nicotine-related disorders | 2012 | 88,369 | 43,145,356 | 204.82 | 208.87 | 0.71 |
| F17 | Tobacco/nicotine-related disorders | 2013 | 91,890 | 44,704,074 | 205.55 | 210.67 | 0.7 |
| F17 | Tobacco/nicotine-related disorders | 2014 | 92,436 | 46,243,211 | 199.89 | 206.03 | 0.68 |
| F17 | Tobacco/nicotine-related disorders | 2015 | 100,145 | 47,760,852 | 209.68 | 217.02 | 0.69 |
| F17 | Tobacco/nicotine-related disorders | 2016 | 102,088 | 49,244,195 | 207.31 | 215.47 | 0.68 |
| F17 | Tobacco/nicotine-related disorders | 2017 | 106,045 | 50,858,679 | 208.51 | 217.28 | 0.67 |
| F17 | Tobacco/nicotine-related disorders | 2018 | 107,602 | 52,431,193 | 205.23 | 213.78 | 0.66 |
| F17 | Tobacco/nicotine-related disorders | 2019 | 109,105 | 54,058,263 | 201.83 | 210.4 | 0.64 |
| F17 | Tobacco/nicotine-related disorders | 2020 | 112,363 | 55,659,365 | 201.88 | 210.79 | 0.63 |
| F19 | Multiple/other psychoactive substance-related disorders | 1999 | 39 | 34,797,841 | 0.11 | 0.09 | 0.01 |
| F19 | Multiple/other psychoactive substance-related disorders | 2000 | 40 | 34,991,753 | 0.11 | 0.09 | 0.01 |
| F19 | Multiple/other psychoactive substance-related disorders | 2001 | 45 | 35,290,291 | 0.13 | 0.11 | 0.02 |
| F19 | Multiple/other psychoactive substance-related disorders | 2002 | 43 | 35,522,207 | 0.12 | 0.1 | 0.02 |
| F19 | Multiple/other psychoactive substance-related disorders | 2003 | 45 | 35,863,529 | 0.13 | 0.15 | 0.02 |
| F19 | Multiple/other psychoactive substance-related disorders | 2004 | 36 | 36,203,319 | 0.1 | 0.1 | 0.02 |
| F19 | Multiple/other psychoactive substance-related disorders | 2005 | 51 | 36,649,798 | 0.14 | 0.15 | 0.02 |
| F19 | Multiple/other psychoactive substance-related disorders | 2006 | 62 | 37,164,107 | 0.17 | 0.19 | 0.02 |
| F19 | Multiple/other psychoactive substance-related disorders | 2007 | 65 | 37,825,711 | 0.17 | 0.16 | 0.02 |
| F19 | Multiple/other psychoactive substance-related disorders | 2008 | 87 | 38,777,621 | 0.22 | 0.2 | 0.02 |
| F19 | Multiple/other psychoactive substance-related disorders | 2009 | 114 | 39,623,175 | 0.29 | 0.28 | 0.03 |
| F19 | Multiple/other psychoactive substance-related disorders | 2010 | 134 | 40,267,984 | 0.33 | 0.34 | 0.03 |
| F19 | Multiple/other psychoactive substance-related disorders | 2011 | 134 | 41,394,141 | 0.32 | 0.3 | 0.03 |
| F19 | Multiple/other psychoactive substance-related disorders | 2012 | 163 | 43,145,356 | 0.38 | 0.39 | 0.03 |
| F19 | Multiple/other psychoactive substance-related disorders | 2013 | 165 | 44,704,074 | 0.37 | 0.35 | 0.03 |
| F19 | Multiple/other psychoactive substance-related disorders | 2014 | 204 | 46,243,211 | 0.44 | 0.46 | 0.03 |
| F19 | Multiple/other psychoactive substance-related disorders | 2015 | 243 | 47,760,852 | 0.51 | 0.5 | 0.03 |
| F19 | Multiple/other psychoactive substance-related disorders | 2016 | 229 | 49,244,195 | 0.47 | 0.44 | 0.03 |
| F19 | Multiple/other psychoactive substance-related disorders | 2017 | 279 | 50,858,679 | 0.55 | 0.53 | 0.03 |
| F19 | Multiple/other psychoactive substance-related disorders | 2018 | 338 | 52,431,193 | 0.64 | 0.64 | 0.03 |
| F19 | Multiple/other psychoactive substance-related disorders | 2019 | 355 | 54,058,263 | 0.66 | 0.64 | 0.03 |
| F19 | Multiple/other psychoactive substance-related disorders | 2020 | 424 | 55,659,365 | 0.76 | 0.72 | 0.04 |

**Supplementary Table 9:** Annual substance-specific mortality rates for deaths involving co-listed COPD and substance-related disorders among U.S. adults aged ≥65 years, 1999–2020.

AAMR indicates age-adjusted mortality rate; SE, standard error. Annual estimates designated “Unreliable” by CDC WONDER were retained for transparency but were not used for Joinpoint trend modeling. Rows with zero or suppressed deaths may not be displayed by CDC WONDER. Substance-specific categories are not mutually exclusive because more than one substance-related ICD-10 code may appear on the same death certificate.

**Abbreviations:** AAMR, Age adjusted mortality Rate; SE, Standard error
