# Supplementary material for: National trends and demographic disparities in mortality involving co-listed chronic obstructive pulmonary disease and substance use disorders among U.S. adults aged ≥65 years: A population-based serial cross-sectional CDC WONDER analysis, 1999 to 2020
Source: Medicine (Baltimore). 2026 Nov 6;105(45):e51448. doi: 10.1097/MD.0000000000051448 (PMC13747554; doi:10.1097/MD.0000000000051448)
Supplement: Supplementary file 9 [file medi-105-e51448-s009.docx]

| **Cohort** | **Lower Endpoint** | **Upper Endpoint** | **APC** | **Lower CI** | **Upper CI** | **AAPC** |
| --- | --- | --- | --- | --- | --- | --- |
| F10 / Alcohol-related disorders | 1999 | 2010 | -2.4582* | -3.3096 | -1.5993 | 0.8433 (0.2778 to 1.4119) |
|  | 2010 | 2020 | 4.6041* | 3.7414 | 5.474 |  |
| F17 / Tobacco/nicotine-related disorders | 1999 | 2005 | 38.5107* | 29.2253 | 48.4633 | 11.5566 (9.1257 to 14.0417) |
|  | 2005 | 2012 | 4.8738* | 1.2569 | 8.62 |  |
|  | 2012 | 2020 | 0.1104 | -1.7547 | 2.0108 |  |
| F19 / Multiple/other psychoactive substance-related disorders | 1999 | 2020 | 10.6108* | 9.6691 | 11.5605 | 10.6108 (9.6691 to 11.5605) |

**Supplementary Table 10:** Joinpoint regression analysis of substance-specific mortality involving co-listed COPD among U.S. adults aged ≥65 years, 1999–2020. An asterisk denotes a statistically significant APC based on a 95% CI excluding zero. Joinpoint modeling was restricted to F10, F17, and F19 because these categories had complete and reliable annual AAMR series from 1999–2020.

**Abbreviations:** APC, annual percent change; AAPC, average annual percent change; CI, confidence interval.
