# Supplementary material for: National trends and demographic disparities in mortality involving co-listed chronic obstructive pulmonary disease and substance use disorders among U.S. adults aged ≥65 years: A population-based serial cross-sectional CDC WONDER analysis, 1999 to 2020
Source: Medicine (Baltimore). 2026 Nov 6;105(45):e51448. doi: 10.1097/MD.0000000000051448 (PMC13747554; doi:10.1097/MD.0000000000051448)
Supplement: Supplementary file 10 [file medi-105-e51448-s010.docx]

| **Year** | **Deaths** | **Population** | **Crude Rate** | **Age Adjusted Rate** | **Age Adjusted Rate Standard Error** |
| --- | --- | --- | --- | --- | --- |
| 1999 | 1261 | 34797841 | 3.62 | 3.61 | 0.1 |
| 2000 | 1301 | 34991753 | 3.72 | 3.73 | 0.1 |
| 2001 | 1250 | 35290291 | 3.54 | 3.53 | 0.1 |
| 2002 | 1227 | 35522207 | 3.45 | 3.42 | 0.1 |
| 2003 | 1238 | 35863529 | 3.45 | 3.47 | 0.1 |
| 2004 | 1169 | 36203319 | 3.23 | 3.2 | 0.09 |
| 2005 | 1244 | 36649798 | 3.39 | 3.41 | 0.1 |
| 2006 | 1237 | 37164107 | 3.33 | 3.32 | 0.09 |
| 2007 | 1121 | 37825711 | 2.96 | 2.98 | 0.09 |
| 2008 | 1197 | 38777621 | 3.09 | 3.11 | 0.09 |
| 2009 | 1246 | 39623175 | 3.14 | 3.15 | 0.09 |
| 2010 | 1317 | 40267984 | 3.27 | 3.29 | 0.09 |
| 2011 | 1389 | 41394141 | 3.36 | 3.33 | 0.09 |
| 2012 | 1433 | 43145356 | 3.32 | 3.34 | 0.09 |
| 2013 | 1652 | 44704074 | 3.7 | 3.66 | 0.09 |
| 2014 | 1811 | 46243211 | 3.92 | 3.85 | 0.09 |
| 2015 | 1979 | 47760852 | 4.14 | 4.08 | 0.09 |
| 2016 | 2108 | 49244195 | 4.28 | 4.19 | 0.09 |
| 2017 | 2312 | 50858679 | 4.55 | 4.43 | 0.09 |
| 2018 | 2635 | 52431193 | 5.03 | 4.95 | 0.1 |
| 2019 | 2770 | 54058263 | 5.12 | 5.01 | 0.1 |
| 2020 | 3403 | 55659365 | 6.11 | 5.91 | 0.1 |

**Supplementary Table 11A:** AAMRs for Death Records Co-listing COPD and Substance Use Disorders After Exclusion of Tobacco/Nicotine-Related Disorders (F17), United States, 1999–2020.

Sensitivity analysis of U.S. adults aged ≥65 years whose death certificates co-listed COPD (ICD-10 J41–J44) and substance use disorders excluding tobacco/nicotine-related disorders (F17). The modified SUD definition included F10–F16 and F18–F19. Neither COPD nor SUD was required to be the underlying cause of death. Crude and age-adjusted mortality rates are expressed per 100,000 population, and AAMRs were standardized to the 2000 U.S. standard population.

**Abbreviations:** AAMR, age-adjusted mortality rate; COPD, chronic obstructive pulmonary disease; SUD, substance use disorder.

| **Segment** | **Lower Endpoint** | **Upper Endpoint** | **APC** | **Lower CI** | **Upper CI** | **AAPC** |
| --- | --- | --- | --- | --- | --- | --- |
| 1 | 1999 | 2009 | -1.8220* | -2.7256 | -0.9099 | 2.1927 (1.3732 to 3.0188) |
| 2 | 2009 | 2017 | 4.6790* | 3.2531 | 6.1246 |  |
| 3 | 2017 | 2020 | 9.5468* | 5.3637 | 13.896 |  |

**Supplementary Table 11B.** Joinpoint regression analysis of mortality rates after exclusion of tobacco/nicotine-related disorders (F17) among U.S. adults aged ≥65 years with death records co-listing COPD and substance use disorders, 1999–2020.

Joinpoint regression was performed as a sensitivity analysis after excluding tobacco/nicotine-related disorders (ICD-10 F17) from the primary F10–F19 substance use disorder definition. The modified SUD definition therefore included F10–F16 and F18–F19. APCs represent the annual percent change within each fitted temporal segment, while the AAPC summarizes the average annual percent change across the entire 1999–2020 period. An asterisk (*) denotes a statistically significant APC based on a 95% confidence interval excluding zero.
