# Supplementary material for: National trends and demographic disparities in mortality involving co-listed chronic obstructive pulmonary disease and substance use disorders among U.S. adults aged ≥65 years: A population-based serial cross-sectional CDC WONDER analysis, 1999 to 2020
Source: Medicine (Baltimore). 2026 Nov 6;105(45):e51448. doi: 10.1097/MD.0000000000051448 (PMC13747554; doi:10.1097/MD.0000000000051448)
Supplement: Supplementary file 11 [file medi-105-e51448-s011.docx]

| **Year** | **Metropolitan AAMR (95% CI)** | **Nonmetropolitan AAMR (95% CI)** |
| --- | --- | --- |
| 1999 | 24.23 (23.66–24.80) | 31.18 (29.85–32.51) |
| 2000 | 36.53 (35.82–37.24) | 51.77 (50.05–53.49) |
| 2001 | 35.15 (34.46–35.84) | 52.31 (50.59–54.03) |
| 2002 | 35.50 (34.81–36.19) | 53.21 (51.47–54.95) |
| 2003 | 80.94 (79.90–81.98) | 124.13 (121.48–126.78) |
| 2004 | 108.00 (106.82–109.18) | 159.97 (156.97–162.97) |
| 2005 | 141.99 (140.64–143.34) | 187.92 (184.69–191.15) |
| 2006 | 148.15 (146.78–149.52) | 193.12 (189.85–196.39) |
| 2007 | 157.75 (156.36–159.14) | 211.21 (207.82–214.60) |
| 2008 | 174.98 (173.51–176.45) | 242.06 (238.45–245.67) |
| 2009 | 165.68 (164.27–167.09) | 230.70 (227.19–234.21) |
| 2010 | 179.00 (177.55–180.45) | 260.05 (256.35–263.75) |
| 2011 | 186.20 (184.73–187.67) | 285.88 (282.02–289.74) |
| 2012 | 189.75 (188.30–191.20) | 302.99 (299.07–306.91) |
| 2013 | 191.91 (190.46–193.36) | 304.75 (300.85–308.65) |
| 2014 | 187.18 (185.77–188.59) | 301.72 (297.88–305.56) |
| 2015 | 196.21 (194.78–197.64) | 323.38 (319.44–327.32) |
| 2016 | 195.15 (193.76–196.54) | 320.19 (316.31–324.07) |
| 2017 | 196.50 (195.11–197.89) | 326.33 (322.45–330.21) |
| 2018 | 192.70 (191.35–194.05) | 326.52 (322.68–330.36) |
| 2019 | 189.05 (187.74–190.36) | 324.74 (320.96–328.52) |
| 2020 | 189.69 (188.40–190.98) | 328.08 (324.32–331.84) |
| **Overall** | **152.03 (151.76–152.30)** | **232.82 (232.08–233.56)** |

**Supplementary Table 7:** Urbanization-stratified age-adjusted mortality rates among U.S. adults aged ≥65 years with co-listed COPD and substance use disorders, 1999–2020

**Abbreviation:** AAMR, Age adjusted mortality rate
